# Experience of rehabilitation management in public hospital after it was identified as designated rehabilitation hospital for COVID-19 patients: A qualitative study

**DOI:** 10.3389/fpubh.2022.919730

**Published:** 2022-07-26

**Authors:** Shuxiao Hu, Changfu Chen, Biwen Yang, Qing Liu, Han Hu

**Affiliations:** ^1^School of Public Policy and Administration, Xi'an Jiaotong University, Xi'an, China; ^2^First Affiliated Hospital, Xi'an Jiaotong University, Xi'an, China

**Keywords:** public hospital, rehabilitation, rehabilitation management, COVID-19, organizational change theory, qualitative study

## Abstract

**Objective:**

It is essential to focus on the rehabilitation of COVID-19 patients after discharge to prevent their long-term sequelae, but there is less research on healthcare organizations enhancing rehabilitation services for patients discharged from COVID-19. Therefore, this study aimed to describe how a public hospital provides better rehabilitation services for patients after being identified as a designated rehabilitation hospital for patients with COVID-19 and attempted to combine the theory of organizational change to analyze how the hospital finally successfully transformed.

**Methods:**

A tertiary public hospital located in the center of Xi'an was selected for the study. It was identified as a designated hospital for the rehabilitation of patients discharged from the hospital with COVID-19. Nine hospital leaders and group leaders closely related to the rehabilitation management work were invited to participate in interviews to explore the fact about the hospital's rehabilitation work. The semi-structured interview with the hospital director and the focus group interview with group leaders were used for data collection. Two researchers independently conducted a thematic analysis of these responses.

**Results:**

One hundred and seventy-eight primary codes, 22 subcategories, six main categories, and one core theme were obtained from data analysis. The main categories include organization and coordination (overall deployment, transfer patient, and external coordination), hospital infection prevention and control (process transformation, ward disinfection, hospital infection training, inspection, and supervision), staff management (staff classification, closed-loop management, and staff health screening), individual services for patients (create an individual scheme, humanistic care, organize special activities, and strengthen communication and guidance), comprehensive supporting (basic medical guarantee, daily necessities guarantee, health and nutrition guarantee, and assistance fund guarantee) and positive transformation (strategic thinking, benchmarking, strengthen cohesion, and expand influence).

**Conclusion:**

The hospital had to transform its operations in the face of a complex environment during the pandemic. After deciding to transform, the hospital effectively prevented nosocomial infections and provided rehabilitation services to 583 patients through systematic management measures such as organizational coordination, staff classification, and personalized services. In the end, it has been successfully transformed and has grown rapidly. To ensure that it can continue to grow sustainably, the hospital enhanced the new ways that have emerged from this transformation.

## Introduction

With the rapid increase in the number of confirmed and dead patients with the COVID-19 (1, 2), the COVID-19 outbreak was declared a pandemic by the WHO in March 2020. COVID-19 has brought huge shocks and changes to all aspects of the global society (3, 4), and all medical departments, including rehabilitation medicine, are no exception (5). For example, as part of efforts to ensure the health system is not overwhelmed, the usual pathway of care in Britain's National Health Service has been suspended (6, 7). Rehabilitation clinicians and programs have had to take on new challenges to provide safe, effective, and efficient rehabilitation for patients recovering from COVID-19 and other traditional rehabilitation diagnoses within the changing circumstances and constraints of this unprecedented epidemic (8).

Rehabilitation is an important part of healthcare and medical management (9). Many patients with COVID-19 have sequelae after being cured and discharged. An Italian study followed 143 individuals 7 weeks after discharge and found that 53% reported fatigue, 43% had difficulty breathing, and 27% had joint pain (10). The NHS England predicted that COVID-19 survivors have high physical, neuropsychological and social needs after discharge (11). Therefore, attention should be paid to the rehabilitation needs of discharged patients with COVID-19. Rehabilitation interventions for COVID-19 patients or those recovering from COVID-19 include aerobic conditioning, strength training, energy-saving training, dyspnea management, and activity-specific training (12, 13). These interventions can improve walking speed, endurance, and pulmonary function syndrome in patients recovering from the severe acute respiratory disease (14, 15). Rehabilitation care can shorten hospital stays at all stages of healthcare, optimize health outcomes and avoid readmissions. Rehabilitation care can also reduce health care and social costs, increase employment rates for COVID-19 survivors, and strengthen the healthcare workforce. Therefore, some recommendations need to be considered to achieve the highest level and quality of rehabilitation services during COVID-19 (and in the long term).

In response to the increasing number of COVID-19 patients worldwide, much of the literature in rehabilitation has focused on the early impact of COVID-19 on the rehabilitation system and on proposing rehabilitation protocols during COVID-19 recovery at the patient level. Many studies have examined changes in their physical symptoms and psychological conditions (16–18). Physical symptoms that COVID-19 patients may experience, such as fever, dyspnea, cough, adverse drug reactions (16), as well as psychiatric symptoms, such as fear of contracting a new virus, loneliness, anger associated with treatment in isolation, and post-traumatic stress (16–18). Comorbidities observed in COVID-19 patients requiring intensive care include muscle weakness, nerve damage, delirium, and more, which have the potential to significantly affect their physical and cognitive functions (19–21). As the pandemic continues to spread around the world, experience with post-discharge rehabilitation care for COVID-19 patients has slowly begun to mature (22).

However, the premise of high-level rehabilitation care for patients with COVID-19 or those who have recovered from COVID-19 is premised on the availability of vectors and adequate resources. The surge in intensive-care patients reduced post-acute facility use, and prolonged hospital stays have put pressure on health systems to consider alternative strategies to promote hospital throughput and maintain capacity (23). These factors have made it necessary to open field hospitals or designated rehabilitation hospitals to treat the increasing number of infected patients. COVID-19 designated rehabilitation hospital is established by the government to fully rehabilitate patients who have recovered from COVID-19. The local government selects a hospital and plans to transform it to receive patients cured of COVID-19 and discharged from the hospital. From the perspective of organizational reform, the public hospital has to make a series of adjustments to adapt to the changes in internal and external environments after being designated as a designated rehabilitation hospital (24). According to the organizational change theory put forward by Kurt Lewin, an organization may experience a variety of differences from change to success due to different factors. However, as a whole, successful organizational change has the common feature that it takes “unfreeze—change—refreeze” to get to success (25).

This study takes a public hospital in Shaanxi Province, China, as an example to describe its experience in carrying out systematic rehabilitation management after it was identified as a designated rehabilitation hospital for discharged patients with COVID-19 during the pandemic. This study also attempts to combine the theory of organizational change to analyze how it finally successfully transformed through its own series of efforts under the complex internal and external environment during that period.

## Materials and methods

### Participants

**Figure 1:**
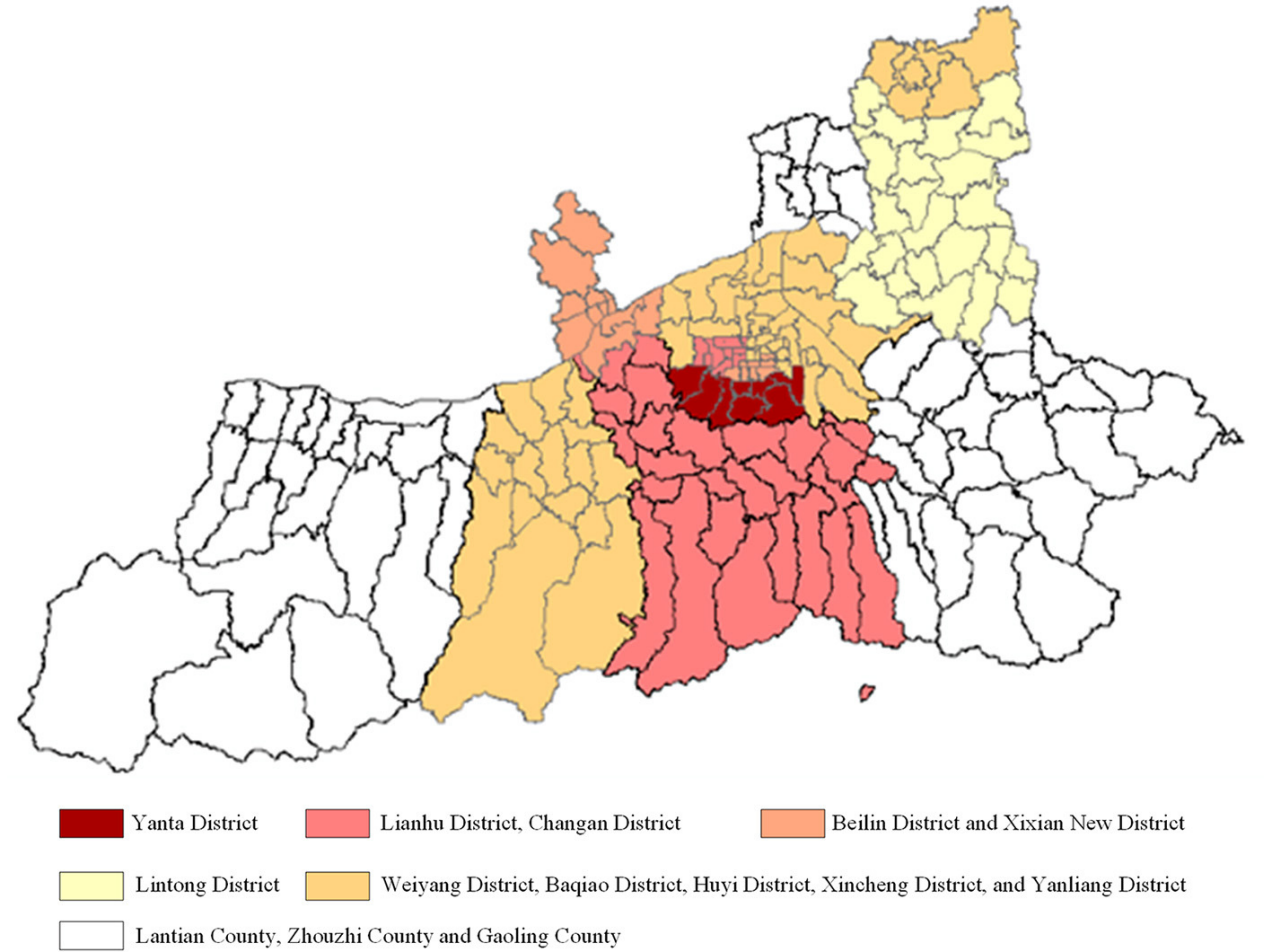
Real-time map of epidemic levels in Xi'an city.

**Figure 2:**
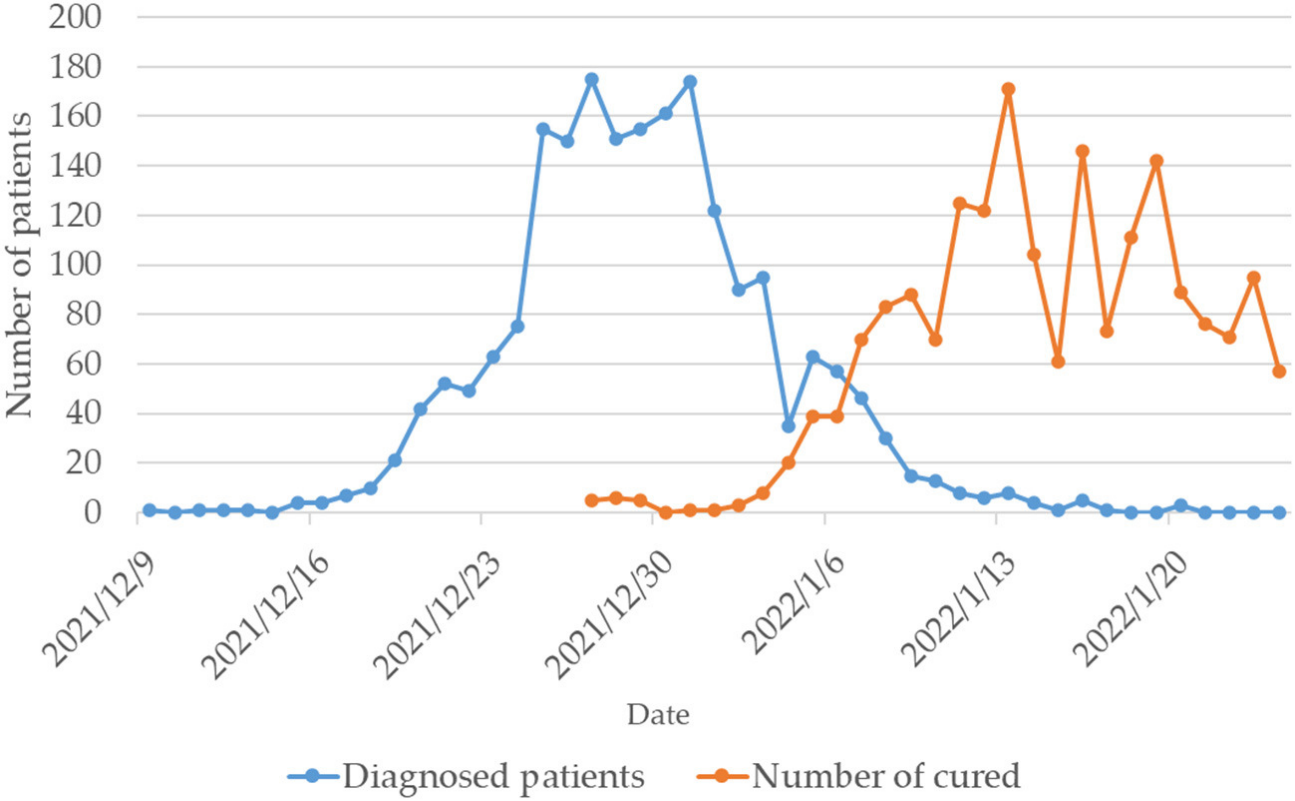
Changes in the number of diagnosed patients and the number of cured in Xi'an during this epidemic.

SXDS Hospital is a general tertiary hospital located in the center of Xi'an City, integrating medical treatment, teaching, scientific research, prevention, health care, and rehabilitation. It was designated as a rehabilitation hospital for discharged COVID-19 patients by Shaanxi Provincial Government on January 6, 2022. From the discovery of the first confirmed patient on December 9, 2021, to the entire city of Xi'an was declared a low-risk area on January 24, 2022, the epidemic in Xi'an lasted 47 days. The epidemic's sever gradually decreases from the city center to the suburbs (Figure 1). The patients with COVID-19 are discharged from the designated hospital after being cured and would be arranged in the designated rehabilitation hospital to receive rehabilitation services. In the late stages of the epidemic, more and more patients were cured and discharged from the hospital with COVID-19. The hospital was identified as a designated rehabilitation hospital for discharged patients with COVID-19 when the number of cured and discharged patients exceeded the number of confirmed patients (Figure 2). A total of 583 discharged patients with COVID-19 were accepted and treated by the hospital during this mission.

The main problem in rehabilitation is that most patients do not need medical treatment but only psychological support and health education. But some patients still need treatment. For this reason, after receiving the task, the hospital held a hospital-wide meeting and arranged for six working groups to complete the task. Each group leader has full authority to handle everything in the working group, accepting feedback from the primary medical staff and reporting the group's work to the director. Each group leader is very familiar with the work they are responsible for. We adopted the method of purposeful sampling to recruit nine hospital leaders and group leaders closely related to this rehabilitation management work (Table 1). The research team obtained the list of working group leaders who had expressed their willingness to participate in the study from the head of the Business Development Department through a formal process with the hospital. Participants were contacted by phone and their consent was reconfirmed before participating in the study.

**Table 1:** Demographic characteristics of participants and data related to rehabilitation management.

**Participant**	**Gender**	**Age**	**Working years**	**Post/department**	**Responsible for the content of this task**
1	Male	62	23	Director of hospital	Mission commander
2	Male	55	20	Vice president of hospital	Patient management
3	Male	52	16	Discipline Inspection Commission	Management of hospital staff
4	Female	53	22	Internal Medicine Department	Nursing team management and hospital infection prevention and control
5	Male	56	18	Anesthesiology Department	Treatment guidelines for patients with disease
6	Female	43	12	Business Development Department	External coordination and Internet projects
7	Female	49	15	Finance Department	Expenditure management of all hospital expenses
8	Male	42	12	Assistant to the hospital director	Nucleic acid test outside the hospital and rehabilitation treatment in the hospital
9	Female	46	21	Health Services Section	Patient admission and discharge management

### Data collection

This study was conducted when the epidemic spread was serious in Xi'an city. The Xi'an municipal government advocates less going out and not gathering. Semi-structured telephone interviews and focus group interviews in the form of video conferences were used for data collection. The day after the SXDS hospital was identified as a designated rehabilitation hospital, the researchers conducted a semi-structured in-depth interview with the hospital director *via* telephone. The interview outline focused on collecting the participants' perspectives, including what preparations have been made in terms of wards, staff, facilities, and equipment after the hospital was identified as a designated rehabilitation hospital? What rehabilitation methods will be adopted for the discharged patients with COVID-19? How will the hospital provide social and psychological counseling for the patients? The whole interview lasted for 90 min. The interviews were recorded verbatim and analyzed as they were conducted to facilitate thematic development.

To saturate the data, the research team conducted online focus group interviews (Tencent conference) with other participants when the epidemic in Xi'an was less severe on January 18. The focus group was mainly composed of group leaders who were responsible for each task, excluding the director and the secretary. On the one hand, focus group interviews without the director and the secretary present allow other managers to speak freely. On the other hand, the focus group interviews can help us verify the credibility of the first telephone interview with the director. The questions of the focus group interview focused on what work did each medical team had done in this rehabilitation management? What effect has the work done on the hospital and patients? How to do an excellent job in the future transformation and sustainable development of hospitals?

When no new information was obtained from the participants, the data reached saturation and the research team canceled subsequent interviews. Data for this analysis was collected from Jan 7 to Jan 18, 2022. The Biomedical Ethics Committee of Xi'an Jiaotong University Health Science Center approved this program (approval number: 2020-1258). Before the interview, all participants were explained the purpose of the study and their roles in the study, and their consent to participate in the research and record their voices was obtained. Participants were assured that the recorded material would be used, but their names and details would not be disclosed. Participants had the right to withdraw at any study stage if they did not want to partake.

### Analysis

The data generated in this study were coded using thematic analysis through a systematic categorization process, and then themes and patterns were identified (26). This approach supports immersion in the data to enable new insights to emerge and inductively develop categories without imposing preconceived categories (27). After the data collection process was completed, all generated data (interviews) were converted to text. After the data was converted, we used Nvivo Chinese software (12.0) to encode important statements.

The general inductive approach was applied in this study (28, 29), allowing our findings to emerge from the most common and dominant themes in the original data without the constraints of more structured methods such as deductive experiments and hypothesis testing studies. The detailed data analysis process is as follows: First, we read the interview text repeatedly to familiarize ourselves with the data and extracted important statements directly related to the research phenomenon. We then formulated broader meanings from important messages while including our premises as closely as possible, similar meanings were clustered into themes, and similar themes were integrated into thematic clusters. In the end, we wrote a comprehensive description of the phenomenon, covering all the revealed themes, and summarized the detailed report into a condensed statement.

To ensure the study's trustworthiness, we applied the criteria of credibility, fit, auditability, and verifiability (30). The principal investigator collected and organized the coded statements followed by discussions with other researchers to derive and refine significant statements, themes, and thematic clusters. In order to establish credibility, two researchers recorded and transcribed the participants' statements verbatim, reviewed the transcripts against the recordings, and finally compared the encoded texts. The *kappa* value of all codes remained between 0.5 and 0.75, indicating a high fit and suitable for data analysis.

## Results

In this study, 178 primary codes, 22 subcategories, and six main categories emerged from the data analysis (Table 2).

**Table 2:** The rehabilitation works organized by the hospital.

**Categories**	**Subcategories**	**Number of materials**	**Reference points**	**Examples of codes**
Organization and coordination	Overall deployment	4	7	• Hold an enlarged meeting to inform the functional departments • Divide into 6 workgroups to work on tasks • Develop plans for infants and young children • The guarantee of funds is handed over to the Federation of Trade Unions for the overall planning
	Transfer patient	2	3	• Mobilize existing hospital patients to discharge • Transfer of inpatients from the hospital to brother hospitals • The process of receiving patients is relatively smooth
	External coordination	3	9	• Connect with the community • Connect with the government • Connect with hotel • Connect with other rehabilitation hospitals to exchange learning experience
Hospital infection prevention and control	Process transformation	3	4	• Divide the infection ward into three zones and two channels • Clear division of clean, semi-contaminated, and contaminated areas • Hard-isolate the patient's channel and the staff's channel • Put anti-epidemic materials on designated floors
	Ward disinfection	2	3	• Cleaned and sanitized the ward overnight • Every paramedic has a sanitization task • The sterilization of other areas in the hospital
	Hospital infection training	1	2	• Training for medical staff • Arrange special personnel to supervise and put on the isolation suit
	Inspection and supervision	1	2	• Daily monitoring of the environment • Nursing is not only to provide nursing services but also some people need to supervise the nosocomial infection
Staff management	Staff classification	2	3	• Divide staff into four categories • Staff classification management for each area • Divide work so that everyone has work to do
	Closed-loop management	4	6	• Don't drop around at work • Do not cross when handover • Closed-loop management of physicians and nurses entering inpatient buildings • Work in the hospital for 6 h, the rest of the time is closed-loop management in the hotel
	Staff health screening	2	5	• Investigate whether to get a COVID-19 vaccine • Health review of blood routine, antibody test, and CT test • Nucleic acid testing for all staff once a day • Rebate personnel at the hotel are double-managed and do a good job of health testing
Individualized services for patients	Create an individual scheme	3	3	• Arrange shifts for patient care needs • Symptomatic treatment of patients with complications or other diseases • Chinese medicine treatment according to the needs of patients
	Humanistic care	3	5	• Purchase daily necessities for patients • Communicate with the heating company to ensure a 24-h hot water supply • Celebrate birthdays for patients • Provide featured catering services
	Organize special activities	3	3	• Encourage patients to get out of bed to exercise • Involve patients in a variety of activities • Create a schedule for patient
	Strengthen communication and guidance	8	10	• Communicate through online diagnosis and treatment on the Internet, • Psychologists are also members of the rehabilitation team to help patients do some counseling • Increase communication by phone or WeChat • Invite psychologists to enter the Internet Hospital
Comprehensive guarantee	Basic medical guarantee	5	10	• Establishment of a new inpatient building as a recovery ward • 7 intensive care units to accept critically ill patients • Clean the ward every day • The medical security team also participates in first aid for critically ill patients • Guarantee common medicines and COVID-19 special medicines • 24 h on duty to enter the ward for rescue • 2/3 of the staff devoted to the care and rehabilitation work
	Daily necessities guarantee	3	6	• Medical staff complete all services for patients • Provide essential household items to patients • Distribute masks to patients every day • Transport items to patients by docking with hypermarkets • Purchase items on third-party platforms
	Health and nutrition guarantee	3	3	• Three meals a day must be nutritious • Patients were guaranteed a bag of milk and a fruit every day • Make sure the patient's food is not cold
	Assistance fund guarantee	3	3	• All treatment funds for patients are paid in advance by the hospital • Including the patient's meal funds, patients do not need to spend a cent • The hospital received no special funds, the hospital pays all in advance
Positive transformation	Strategic thinking	3	5	• The whole hospital attaches great importance to this task • Take this opportunity to shorten the relationship with the government, • Take this opportunity to do a good job in the rectification and transformation of the hospital • The original 5G + Health Management project will be done well
	Benchmarking	2	2	• Learn from the industry's outstanding benchmarking units • Communication with Qinhuang Hospital on the patient management model
	Strengthen cohesion	4	6	• Strengthen confidence in video conferences for middle-level leaders • Employees actively participate in this task • Everyone's hearts are connected in special times • Hospital team cooperation and dedication are reflected
	Expand influence	2	6	• Services should also be provided after 14 days of discharge • Extend online services to patients through an internet hospital platform • Follow up with patients and carry out targeted interventions

**Figure 3:**
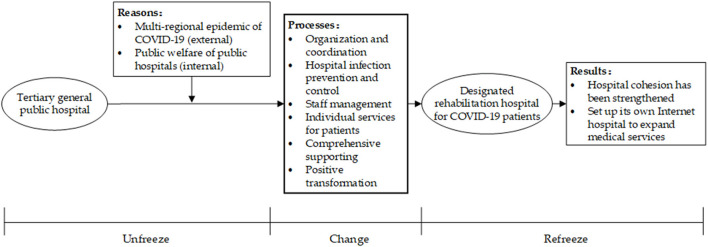
The theoretical framework of the organizational self-transformation process.

Based on the above coding conceptualization of the interview data, the distillation of the categories, and the summarization of the themes, we used the theoretical coding strategy of “cause, process, and effect” from Glaser's classical grounded theory to summarize them into the core theme of “Self-transformation of public hospital after it was designated as COVID-19 rehabilitation hospitals.” This study also combines a planned organizational change model proposed by Lewin, which contains three steps of unfreezing, changing, and refreezing. A theoretical framework was built for the public hospital self-change process (Figure 3) to explain and guide how public hospitals launch, manage and stabilize the organizational change.

### Organization and coordination

#### Overall deployment

After receiving the notification from the superior that it was determined to be the designated hospital for the rehabilitation and treatment of COVID-19 cured patients, SXDS Hospital quickly made various preparations and entered a state of “24-h standby preparation” that can welcome patients at any time. Hospital leaders systematically thought about and discussed this task that day, discussing how to take over the battle against the pandemic and how to fight this battle well. The next morning, the hospital mobilized all-party committee members and relevant functional departments by holding a party member meeting. After discussion, a preliminary action plan was determined, and a plan was prepared for some emergencies.

*On the morning of the 7th, we held an enlarged meeting of the Party Committee of the Academy, and through the Party organization, we communicated this matter to our Party committee members and relevant functional departments*. (Participant 1)

In the process of playing the role of a rehabilitation hospital for patients cured of COVID-19, SXDS Hospital also divided corresponding task groups according to different business contents and arranged special personnel to be responsible for the leadership of sub-tasks, and achieved efficient operation in management.

*Our hospital is divided into six task groups, of which I am in charge of the support group. My support group has five functional departments—Finance, General affairs, Information, Medical equipment, and Pharmacy*. (Participant 7)

#### Transfer patient

After SXDS hospital was identified as a designated rehabilitation hospital, it was necessary to transfer all of the original inpatients out of the hospital in order to prevent them from becoming infected. Therefore, the middle-level leaders of the hospital did their best to explain and comfort the patients as well as their families and took the initiative to push the patients to the ambulance, pack up the items for the patients, and assist the family members of the discharged patients from the hospital, etc. For the critically ill patients who were originally hospitalized, the hospital contacted other medical units on a humanitarian basis to request they accept these patients. After all these inpatients were transferred, the hospital could re-plan the ward. In addition, at the time of transferring the original inpatients, the hospital has also achieved a seamless connection in receiving COVID-19 inpatients based on the work plan formulated the previous day.

*On January 7th, we have another important task, which is to transfer all the patients in our hospital. As of 10 p.m., we have successfully transferred all 250 patients out*. (Participant 1)

*In the process of picking up patients, due to our adequate preparation and smooth communication, after the patient arrived at our hospital, there was not a single patient who stayed in the hospital and was not arranged*. (Participant 2)

#### External coordination

In the process of SXDS hospital serving the cured patients with COVID-19, the hospital was not coping alone but also connected with many stakeholder groups. For example, the hospital was identified as a designated rehabilitation hospital for COVID-19 patients by the government. Therefore, it must first coordinate with the government to successfully complete the task. Secondly, it is necessary to conduct learning exchanges with other designated rehabilitation hospitals and negotiate the statistics of patient information. In addition, when the cured patients reach the standard of recovery and discharge, the hospital also needs to communicate with the community to ensure that the cured patients can return home smoothly.

*The task assigned to us by the Xi'an Epidemic Prevention and Control Headquarters, we have to communicate with it. After the communication, we have to complete how to communicate with the government*. (Participant 2)

*In order to ensure that patients can be discharged from our hospital smoothly, we have set up a working group for this discharge and community connection*. (Participant 9)

### Hospital infection prevention and control

#### Process transformation

During the epidemic outbreak in Xi'an, nosocomial infections occurred in three designated hospitals in Xi'an. Therefore, another critical task of SXDS hospital is to prevent nosocomial infection. In this regard, hospital managers have thought of a lot of ways, such as transforming the hospital from a large process so that the hospital's admitted patients and staff can walk in different areas, and the patients and staff are not in the same area, which can effectively prevent the spread of diseases.

*Actually, one of our characteristics is that the infection ward built on the first floor in the past was divided into three areas and two passages. We made it three-dimensional and created a three-dimensional division of three regions and two passages*. (Participant 2)

*Then on the evening of January 7th, we clarified the division of clean areas, semi-contaminated areas, and polluted areas, which is a very critical link in terms of nosocomial infection*. (Participant 1)

#### Ward disinfection

Sorting out and sterilizing the ward is the essential preparation for the hospital to carry out rehabilitation and treatment tasks. The hospital will focus on arranging for nursing staff to be responsible for implementing this work.

*Every person who enters the building has a disinfection task, not only to take care of patients, to do basic nursing and nursing work but also to disinfect*. (Participant 2)

*We cleaned and sanitized the ward overnight, which is the essential preparation for the ward*. (Participant 1)

#### Hospital infection training

In order to effectively control the occurrence of nosocomial cross-infection, the hospital has also continuously carried out special training on epidemic prevention and control for all medical staff in the hospital. The focus is on strengthening the learning of knowledge related to epidemic prevention and how to do personal protection at work. This effectively improved the medical staff's awareness of hospital infection prevention and control, standardized the daily work process, and ensured that the hospital's prevention and control work was carried out in an orderly manner.

*And then there is our hospital infection training for medical staff. Especially the training on putting on and taking off isolation suits and protective suits*. (Participant 2)

*If the method of taking off the isolation suit is wrong and incorrect, it is easy to cause one's own infection, so this procedure is very critical*. (Participant 2)

#### Inspection and supervision

In order to continuously strengthen infection management in hospitals, implement various infection control measures in each department, and ensure that all staff, the whole process, and the whole hospital strictly adhere to the bottom line of “zero infection,” the hospital has also strengthened its inspection and supervision functions to prevent and control the occurrence of nosocomial infections to the greatest extent possible.

*In addition, we have to monitor the environment every day. Judging from the operation for more than 10 days, our environmental monitoring is all negative. It should be said that we are still relatively stable at present*. (Participant 2)

*We stimulated all the head nurses and infection controllers as our supervisors to strengthen our hospital sense supervision, and the purpose is also to prevent the occurrence of hospital sense*. (Participant 2)

### Staff management

#### Staff classification

Although the main business of the SXDS hospital has changed after being identified as a designated rehabilitation hospital for COVID-19 patients, the work of the opened Internet hospital, or the service work for the isolation hotel, still requires people. Therefore, during this period, the hospital carried out a work diversion so that everyone could do an excellent job in the various tasks at hand.

*We divided our staff into four categories. The first category is people who enter the COVID-19 ward and have direct contact with patients, and the second category is security service personnel, who do not have direct contact with patients but may also enter the vicinity of the rehabilitation ward. The third category is those who are not physically suitable to work in the hospital, such as those who are pregnant or breastfeeding, and a reasonable arrangement should be made for them. The fourth category is the work that needs to be managed while stationed in isolated hotel personnel*. (Participant 3)

#### Closed-loop management

After classifying the personnel, the hospital adopted closed-loop management for some personnel, especially the doctors, nurses, and service personnel who entered the inpatient building, and provided room and board for the closed-loop management personnel. The staff who are arranged to work in the hotel also adopt a closed-loop management method. In addition, the hospital sense department has specially formulated rules and regulations for closed-loop management.

*The meeting the day before was the target of adjustment. Previously, we thought that the staff who entered the ward were the main targets of strict closed-loop management, but the municipal government required all hospital staff the night before yesterday to be regarded as the target of crucial supervision*. (Participant 3)

*In addition to working in the hospital, the medical staff works 6 h a day, and the other time is closed-loop management in the hotel. In the hotel, it is also a single room, and they are not allowed to visit each other and are not allowed to gather*. (Participant 2)

*We entered 585 people in these 12 wards, 300 medical staff, and 180 cleanings and security personnel outside the building. These personnel is also closed-loop, and they need the hospital to provide accommodation*. (Participant 7)

#### Staff health screening

A general goal at the hospital level is zero infection, so in addition to closed-loop management of classified personnel, another job the hospital does in personnel management is to monitor personnel health. Especially for this group of people who have direct contact with patients, before such personnel is stationed, in accordance with the requirements of the National Health and Health Commission and the Prevention and Control Headquarters, all personnel must undergo a physical examination and pass the physical test before they are allowed to work. If there is a problem with the inspection, the hospital will also make corresponding adjustments.

*We first conducted an epidemiological investigation in the early stage. Whether the new crown vaccine has been vaccinated in the whole process is the most basic*. (Participant 3)

*There is also the nucleic acid test of the staff in our hospital. The nucleic acid test of all staff is carried out once a day*. (Participant 2)

### Individual services for patients

#### Create an individual scheme

After the clinical cure of COVID-19 patients, there are often residual problems in breathing, appetite, sleep, psychology, etc. It may also be because the illness aggravates or affects the original underlying diseases. Therefore, to better promote the recovery of patients after the cured patients enter the recovery period, the hospital makes a comprehensive evaluation based on the overall condition of each patient's disease process, such as symptoms at onset, treatment plan, length of recovery time, previous underlying diseases and the patient's ability to live in daily life, etc. Then determine an individual rehabilitation treatment plan.

*Then we also formulated some nursing routines and nursing regulations for this patient in our hospital and arranged shifts*. (Participant 4)

*If there is a need for some patients during the recovery period, we can treat them with traditional Chinese medicine according to the needs of the patients. If some patients have poor cardiopulmonary function, such as the elderly who need oxygen, we will also provide some oxygen therapy*. (Participant 1)

*From waking up in the morning to resting in the evening, when to wash, eat, and exercise, we will formulate a model for the patient. Of course, the patient can do it without following the schedule when there are individual needs*. (Participant 9)

#### Humanistic care

In addition to physical health, the hospital also pays attention to the mental health of the recovered patients in a timely manner, allowing them to feel humanistic solid care during the recovery period. For example, the hospital tries its best to give more care to the patients in life and actively helps the patients to purchase daily necessities. When doing catering, the hospital also provided special services, including baby meals, children's meals, and diabetes meals.

*We will ask all medical staff to give them some humanistic care. After all, the patient has to stay in the hospital for more than 10 days. From a closed area to a closed area, the patient may have some psychological pressure or other negative emotions*. (Participant 1)

*We are now trying to make our doctors be more careful, be more careful than usual when treating patients, speak softer, have a better attitude, and soothe the patient's emotions*. (Participant 4)

#### Organize special activities

Recovered patients are most of the time under closed management during treatment in the hospital. To help them expand their interpersonal circle, the hospital has explored various forms of activities. For example, as the Chinese traditional Spring Festival was approaching, patients were given laba porridge and warm wishes on the laba Rice Porridge Festival. In addition, the hospital celebrated birthdays for some patients and invited others to participate. Through these activities, patients feel solid humanistic care. These activities also help them to recover quickly.

*We have adopted various forms of activities so that everyone can participate in it, and it is more pleasant to enjoy the treatment process for more than 10 days*. (Participant 1)

*Next, we will celebrate the patient's birthday with the nursing team and provide warm services*. (Participant 7)

#### Strengthen communication and guidance

The discharged patients with COVID-19 were transferred to the designated rehabilitation hospital for continued rehabilitation after regular treatment at the designated treatment hospital. The mindset of patients is different in these two hospitals. Patients infected with COVID-19 were more obedient to the hospital when they received service at the designated treatment hospital because they felt they were a patient. However, when patients were cured and discharged from the hospital to receive rehabilitation services at the designated rehabilitation hospital, they thought of themselves as no longer patients and were less obedient to the management. Facing the negative emotions of patients, the hospital first adopted various methods to strengthen communication and gain the understanding of patients.

*We have also thought of a lot of ways. For example, we can set up a WeChat group of doctors, nurses, and patients, and patients can express their opinions in the WeChat group*. (Participant 4)

*We are a medical institution, and we will not provide them with such good services as hotels do. We also communicate with patients in some ways, and after communicating well, we gain an understanding of the patients*. (Participant 2)

By medical standards, recovered patients are cured, but the way they behave, and the way they see others acting, is a reminder that the true cure has not yet been achieved. Therefore, for patients' physical and mental recovery, the hospital has also taken practical interventions to help COVID-19 patients regain their enthusiasm for life and work so that they fully realize that they have not been abandoned by society.

*Our hospital launched an online psychological consultation, and if the patient had any psychological problem, our online chief physicians were also constantly explaining it*. (Participant 1)

*We are still doing this online psychological counseling service, and we invite some psychologists from other hospitals to settle in our Internet Hospital to provide timely psychological counseling and intervention for recovered patients*. (Participant 5)

*The psychologist is also a member of the rehabilitation team and conducts some communication, exchange, and mediation on the patient's psychological problems*. (Participant 6)

### Comprehensive supporting

#### Basic medical guarantee

In order to ensure the smooth progress of medical treatment, rehabilitation treatment for COVID-19 patients, and first aid for critically ill patients, the hospital has given full play to the advantages of various human and material resources based on the innate conditions of the original general hospital. It provides the most basic medical security conditions for patients who have recovered from COVID-19, such as setting up a special medical security team to be responsible for this work, opening additional wards to accept critically ill patients, and ensuring the use of essential conventional and special drugs for patients.

*In addition, our hospital is a general hospital, we have a relatively good condition, that is, we can still keep up with the treatment of patients' basic diseases*. (Participant 2)

*In addition to supplies, we also have to ensure the medicines we give to patients. In terms of drugs, we must find ways to ensure regular drugs and special medicines for COVID-19*. (Participant 1)

*So, two-thirds of our staff are involved in the treatment and recovery of the COVID-19 epidemic*. (Participant 7)

#### Daily necessities guarantee

In the case of comprehensive material support under the epidemic, it is necessary to establish a team that responds quickly. The team's ability to cooperate is essential. Among the six departments divided by SXDS hospital for this task, the comprehensive material support team is one of them. They created a special response team. In the material group for prevention and control, members in the group respond immediately, and they can perform tasks well. Through various efforts, group members have provided comprehensive living supplies to patients who have recovered from COVID-19.

*Actually, we are also considering that we want to connect with a superstore and then transfer it directly to the patient*. (Participant 1)

*These patients basically do not receive medical treatment. The primary need is daily necessities, from nail clippers to razors. We collect and purchase on behalf of them and use the fastest and most convenient way of shopping to meet their needs for daily items*. (Participant 7)

#### Health and nutrition guarantee

A good diet is essential for curing patients. Drug treatment blocks the replication of the virus, and immunity is the final line of defense to kill the virus and invade cells. In terms of catering services, the hospital has carried out nutrition matching for three meals to ensure patients' essential diet and nutrition during hospitalization and enhance immunity.

*There is also the issue of food and beverages. Three meals a day must be nutritious and delicious*. (Participant 2)

*For the quality standard of meals, we also ensure that patients can have a certain amount of nutrition here three times a day, such as a bag of milk every day and a fruit every day. We must guarantee these things to patients*. (Participant 1)

*You know that for 585 patients' three meals a day, we must ensure that the meals are not cold, and we must ensure that 585 meals are delivered within 40 min. Everyone is under a lot of pressure, and we have to take the risk that we will not be infected. Therefore, our hospital director set up the delivery group and the transfer group in time*. (Participant 7)

#### Assistance fund guarantee

Since SXDS Hospital was identified by the government as a designated rehabilitation hospital for the recovery of COVID-19 patients this time, it was a process of “receiving orders in a critical situation,” and the hospital did not receive special funds in advance. All the funds used for medical assistance this time were paid in advance by the hospital. The chief accountant has done a lot of work in this area to raise funds.

*All the relief funds we give to the patient, including his catering funds, are all paid in advance, and the patient does not need to spend a penny*. (Participant 2)

*We didn't get the special funds, and we didn't apply for it ourselves. All of them were paid by the hospital itself. It can be said that we were “instructed in times of crisis” and unconditionally accepted this task*. (Participant 1)

### Positive transformation

#### Strategic thinking

Being identified as a designated rehabilitation hospital for COVID-19 patients this time has the triple attributes of politics, social welfare, and self-reform for the hospital. Hence, the whole hospital attaches great importance to this. Strategically, the hospital regards it not only as a high-quality political task delivered by superiors but also as a task of serving the masses and anti-contribution to society. At the same time, it also regards it as a way for the hospital to realize self-reform and transformation.

*Our hospital has become a rehabilitation hospital through this transformation, it should be said that the relationship between the government and us will be stronger*. (Participant 2)

*We hope that after completing the rehabilitation and treatment of COVID-19 patients with high quality and efficiency, we will continue to “strike while the iron is hot,” gather the energy of all hospital staff, and do an excellent job in the follow-up transformation and development of the hospital*. (Participant 1)

#### Benchmarking

While taking on this task and making a series of decisions, the hospital conducted a systematic review, profound reflection, and experience summarization of the past management, and had the courage to face up to and admit the many deficiencies and mistakes in the past management, and learned the lessons of these past failures. At the same time, it also actively learned from the excellent benchmarking units in the industry.

*Then before this patient was admitted, we also did a lot of work in nursing. For example, we communicated with Qinhuang Hospital, and we also asked about a model of managing these patients*. (Participant 4)

#### Strengthen cohesion

The development of the hospital is inseparable from the hard work of every employee. The hospital's task of accepting this task belongs to “being ordered to be in crisis.” The time is very urgent. It is by no means that one person or a group can do all the preparatory work. It is the active support and decisive actions of all the staff to promote it. The rapid and effective implementation of various measures of the hospital.

*We didn't actually do a lot of mobilization in the early stage, but the employees could actively participate in this task, which moved me very much*. (Participant 1)

*It is the setting of our entire hospital to face the COVID-19 rehabilitation designated hospital this time. Under such opportunities and challenges, our hospital has grown, and our team cooperation ability and dedication have been reflected*. (Participant 6)

*Because as a member of the party committee, I also constantly boost the morale of everyone in our group, including conveying the spirit of the superiors to everyone, complimenting their work, and fighting with everyone*. (Participant 7)

#### Expand influence

Coinciding with the new year when this task was undertaken, the hospital leadership team hopes to take this opportunity to do an excellent job in the rectification and transformation, and development of the hospital. For example, as a regional urban medical group, the hospital makes full use of the advantages of 5G Internet Hospital and Internet of Things technology to carry out the Internet + Health Management service for COVID-19 patients and discharged patients on the Internet platform. This makes the patient feel that although he has left the hospital, the service can still be extended to the patient through the Internet.

*I plan to set up an online rehabilitation and health management class on the Internet. On the Internet Hospital platform, online health management services for patients with COVID-19 on the Internet will be launched*. (Participant 6)

*After the patient is discharged from the hospital, how can we give him some health guidance in the next step? How can we make this patient in a radius of several kilometers recognize our hospital? For example, he will come to our hospital for the following review. So that's the things we're going to consider doing next*. (Participant 9)

## Discussion

The rehabilitation of patients with COVID-19 represents a new clinical and organizational type of rehabilitation. From the clinical viewpoint, rehabilitation of COVID-19 patients requires special medical assistance, and these patients have higher diagnostic and therapeutic needs than non-COVID-19 rehabilitative patients (31–34). The planning of the COVID-19 recovery unit requires an organizational analysis of the specific needs of this new clinical entity, which has not been previously provided by healthcare organizations around the world (35). A comprehensive health organization is essential to respond to current and future epidemics. It must include strengthening and properly organizing rehabilitation as an integral part of the treatment process. Figure 3 is an action framework for transforming public hospitals constructed from the interview data combined with the theory of organizational change. It was used to analyze how a public hospital carried out systematic rehabilitation management after it was identified as a designated rehabilitation hospital for discharged patients with COVID-19 to successfully treat patients and realize its own transformation. This is of great significance for improving the operational efficiency of medical institutions and the allocation of medical and health resources, as well as improving the quality of patient rehabilitation services under the pandemic.

### Changes in the internal and external environment drive the hospital to unfreeze

The delta virus in this epidemic in Xi'an has the characteristics of “fast spread,” “high viral load,” and “occult transmission” (36). It has formed a certain scale of community transmission and spillover cases. Yanta and Beilin districts, which are located in the city center, were the areas of high epidemic prevalence (Figure 1). Coincidentally, SXDS hospital is also located in the center of Xi'an and is a general tertiary hospital that integrates medical treatment, teaching, scientific research, preventive care, and rehabilitation. It is equipped to treat patients recovering from COVID-19. Changes in the external environment and internal conditions make it a designated rehabilitation hospital. Its original routine medical consultation services had to be suspended and turned into rehabilitation services for patients discharged from the COVID-19. That is, the organization began to unfreeze.

The focus of this step of organizational unfreezing is to create motivation for change. Employees are encouraged to change their old behavior patterns and work attitudes and adopt new behaviors and attitudes that are adapted to the organization's strategic development (37, 38). In order to do this, the hospital strategically treats it as both a political task that must be accomplished and a social task that serves the public, as well as an internal task to achieve self-reform. In other words, this task has the triple attributes of politics, social welfare, and self-reform for the hospital, so the whole hospital attaches great importance to it. After the hospital received the government's notice to become a designated rehabilitation hospital, a hospital-wide staff representative meeting was held to inform all-party committee members and relevant functional departments. This made the leaders and staff realize the urgency of the change. The most important thing is that the hospital had developed a preliminary action plan through this congress.

### The hospital carries out systematic rehabilitation management to actively transform

The complex internal and external environment changes have brought opportunities and challenges to this hospital. Timely adjustment, improvement, and innovation of elements in the organization, such as its management philosophy, working methods, organizational structure, staff management, organizational culture and technology, largely determine the success of a change (39). Through semi-structured in-depth interviews and focus groups with senior executives of the public hospital, this study provides essential information about the successful transformation of a public hospital after being identified as designated rehabilitation hospital. This study confirmed the effectiveness of a series of transformation efforts, such as renovating wards to meet the treatment standards of COVID-19 rehabilitation hospitals and providing special rehabilitation services for patients, in the face of multiple challenges such as lack of funds, prevention of nosocomial infections, and psychological panic among the population.

First, SXDS hospital adopted an internal and external organizational coordination after becoming the designated rehabilitation hospital for the COVID-19. In China's national conditions, public hospitals are subordinate to government administrative agencies. They are subject to administrative mechanisms and their governance means have administrative characteristics (40). Therefore, being identified as a designated rehabilitation hospital for COVID-19 also has administrative tasks for public hospitals. This study shows that organizational coordination inside and outside the hospital can help organizations quickly complete basic deployments after unfreezing. After SXDS Hospital was identified as a COVID-19 rehabilitation hospital during the epidemic, the hospital managers strengthened external contacts at the first time, and they quickly established a task group. This is the primary element of China's modern emergency management system with “three cases and one system” as the core for public health emergencies since the SARS (41). In extraordinary times, the hospital implements a significant leadership responsibility and accountability system by setting up a working leadership group or task force to avoid confusion of authority and responsibility and multiple leadership.

In addition to ensuring medical treatment, another significant challenge of becoming a rehabilitation hospital for COVID-19 patients is to prevent nosocomial infection. From the national incidence rate, approximately a quarter of medical staff are direct victims of infectious diseases (42). These results appear to be that participants in the study contracted the virus early in the outbreak before disinfection systems were firmly established. In addition, this suggests that the problems that emerged in past outbreaks, such as inadequate public health crisis response systems, limited understanding of the outbreak, and poor communication about disease risks, remain (43, 44). The study reveals that a “three districts and two channels” process transformation of the existing hospital infrastructure is necessary to prevent nosocomial infections. The “three districts” refer to clean, contaminated and semi-contaminated areas. The “two channels” refer to the medical staff channel and the patient channel. The coronavirus spreads from person to person through a droplet and aerosol transmission and indirect transmission by touching pollutants or contaminated surfaces (45). The process transformation of three districts and two channels separates patients from susceptible groups. It reduces the risk of infection by physically achieving not being in the same area, not meeting, and not crossing. In addition, ward disinfection, hospital training and inspection and supervision are also essential. In fact, the hospital had not experienced a single nosocomial infection during the period when it was designated a rehabilitation hospital for patients with COVID-19.

Personnel management in public hospitals is the crucial and difficult point of becoming a designated rehabilitation hospital. The risk of coronavirus infection among medical staff, especially primary care doctors and nurses, is very high (46). Personnel management should not allow mistakes. Otherwise it will cause large-scale nosocomial infections and increase the pressure of COVID-19 treatment. Considering the different aspects of the task content during an epidemic, the hospital has categorized and managed its personnel. Personnel involved in direct contact with patients, security service personnel, personnel who are not suitable to work in the hospital, and management personnel of the isolated hotel, each perform their duties to improve work efficiency. The requirement for staff to maintain physical and social distance, wear masks, closed-loop management, vaccination, and pre-service health screening are infectious disease prevention requirements aimed at reducing the probability of nosocomial infection events (42), as well as out of the need to protect health care resources. Medicine has the characteristics of solid professionalism and a long personnel training cycle. Medical personnel is scarce medical and health resources. Especially during the COVID-19 epidemic, countries may face the shortage of medical resources. These requirements for staff can help protect themselves and patients and reduce the probability of being infected.

Another critical point for the success of hospital transformation involves the rehabilitation services and various guarantees provided by the hospital. The purpose of designing a designated rehabilitation hospital is to effectively provide a high level of rehabilitation services to patients in a specific area so that patients can be sent to their homes smoothly. In this sense, taking effective rehabilitation measures to promote the smooth recovery of patients is the most fundamental task of designated rehabilitation hospitals. This study reported that the hospital had explored effective measures to recover the patient's smooth recovery. The coronavirus causes various degrees of damage to patients' lungs, kidneys, heart, and other organs, and even multi-organ failure (47, 48). During the physical rehabilitation phase, exercise rehabilitation, as one of the main methods of pulmonary rehabilitation training, is of great significance to the improvement and rehabilitation of patients with acute and chronic lung injury (49). Moderate physical exercise can promote blood circulation and allow immune cells to transport and destroy viruses in the body in time, which is the primary basis of physical exercise to effectively resist and contain coronavirus (50). Therefore, in the context of the COVID-19 pandemic, physical activity may help to positively regulate the immune system and improve physical and mental health. This rehabilitation hospital puts physical exercise at the top of its rehabilitation program. Furthermore, nutritional support is critical together with rehabilitation to improve the chances of recovery for COVID-19 patients (34). In order to ensure the nutrition of patients, the hospital has made appropriate arrangements for three meals a day.

Both the widespread contagion and the lockdown inevitably affected the psychological changes in the population (51–53). This was also a significant challenge for the hospital. Various frailty and dysfunction caused by COVID-19, as well as activity restrictions and decreased ability to live and participate in society caused by the disease, making convalescent patients often suffer from anxiety, depression, panic, insomnia, and many other psychological problems. Previous studies have shown that patients with COVID-19 experience a significant mental burden during their recovery (54, 55), and new models to mitigate these effects may help reduce this severity. This study confirms the need to assess the consequences of the pandemic over time for people who suffered functional limitations prior to COVID-19, as their physical and mental status may be altered by the pandemic, particularly as a result of lockdown (56). This rehabilitation hospital and its staff have eased patient uncertainty by providing effective COVID-19-related information and emotional support. More importantly, the rehabilitation hospital adopted practical and effective intervention measures to timely identify the psychological needs of patients. According to different genders, occupations, ages, and groups, the hospital carried out different levels of psychological rehabilitation strategies such as in-hospital intervention, telephone counseling, and online consultation. For pregnant women, the elderly, children, the disabled, and other special groups, the hospital took into account their special psychological state and psychological needs, formulated targeted psychological rehabilitation programs, provided comprehensive psychological rehabilitation support, and carried out personalized psychological rehabilitation guidance.

The success of the designated rehabilitation hospitals also depends on whether there is sufficient financial support. First, the spatial layout and negative pressure ventilation system of the ward buildings of the designated rehabilitation hospitals must meet the requirements for preventing and controlling respiratory infectious diseases. The renovation of these facilities requires the hospital's funds to be guaranteed. Second, the closed-loop management adopted by the hospital for its staff is carried out at the hotel. These staff members are required to bear their own costs for accommodation and food. Third, medical expenses incurred by the patients with COVID-19 are subsidized by the government after basic medical insurance, primary medical insurance, and medical assistance are paid in accordance with the regulations (57). In fact, these expenses are usually prepaid by designated rehabilitation hospitals and then financially subsidized by the government to the hospital, but there is often a lag in time. In this study, the hospital's chief accountant did a lot of work to raise funds. However, if government financial subsidies are not available, the epidemic continues to spread, and the number of rehabilitated patients continues to increase, this may lead to the unsustainable operation of the designated rehabilitation hospital.

### The hospital refreezes itself after successful transformation

During the refreeze phase, the necessary reinforcement means are needed to fix the new attitudes and behaviors and to stabilize the organizational change (25). In the process of providing rehabilitation services for patients, this hospital has also achieved rapid growth and pointed out the direction for its subsequent transformation and development. There is ample evidence that COVID-19 has increased the need for recovery not only in patients with COVID-19 but also in patients with sequelae of long-term illness or disability (58). Therefore, during the COVID-19 pandemic or any other similar outbreak (59), rehabilitation services should continue to be provided, combined with medical management of different stages of acute, subacute, and long-term COVID-19 (60). This study shows that the hospital has achieved good results by continuing to serve patients through Internet diagnosis and treatment, which can attract some of its previous patients to its Internet hospital for online consultation and disease guidance. Due to the contagious nature of COVID-19, expert consensus-based guidelines recommend limiting direct contact between therapists and COVID-19 patients. Telemedicine can be used as electronic personal protective equipment (PPE) to reduce the risk of exposure and contamination for patients and practitioners (61). Telerehabilitation is defined as “the provision of rehabilitation and rehabilitation services through information and communication technologies. … Telerehabilitation services may include assessment, assessment, monitoring, prevention, intervention, supervision, education, counseling, and guidance” (62). Therefore, the hospital took advantage of the role of the designated rehabilitation hospital for patients with COVID-19 to transform and carry out other services of Internet + Diagnosis and Treatment, providing medical and drug distribution services for more patients during the recovery period, even meeting the medical needs of patients with other diseases, and realizing the sustainable development of the hospital itself.

### Limitations

This study is not without its limitations. First, due to COVID-19 movement restrictions, interviews were conducted *via* phone and Tencent Conferences online. This data collection may have limited our in-depth exploration of participants' non-verbal expressions. Second, although the sample of this study has a certain purpose, the sample size is relatively small, and the sample size can be expanded for further research in the future. Third, since the subjects of this study are Chinese hospitals and their managers, the conclusions can be generalized to rehabilitation institutions in China but may not be generalized to other countries due to differences in cultural conditions.

Despite this, this study still has essential significance. Based on the “Unfreeze-Change-Refreeze” theory of organizational change, it provides in-depth evidence for how rehabilitation hospitals can provide high-quality rehabilitation services to patients and achieve their own successful transformation with limited resources during a pandemic. This study raises awareness of the need to improve the operation and management of rehabilitation hospitals to improve the “public welfare” of public hospitals and to develop relevant interventions.

## Conclusion

This study aims to understand how a public hospital can provide high-quality rehabilitation services for patients after being identified as a designated rehabilitation hospital for patients with COVID-19 and realize its own transformation. This study analyzes the many challenges of the hospital that had to be unfrozen in the face of a complex internal and external environment from the perspective of organizational change. After unfreezing, the hospital has actively transformed through organization and coordination, hospital infection prevention and control, staff management, providing individual services for patients, and comprehensively supporting. The hospital effectively prevented nosocomial infections and successfully cured 583 patients with COVID-19 through the above management. The most important thing is that the hospital has developed itself rapidly after this transformation. Through the social reputation accumulated during this mission, the hospital established an Internet Hospital to expand medical services to more residents. The hospital staff also became more united after this mission, and the hospital continued to run a public hospital that satisfied the people with the help of this cohesion, which means that the organization has been refrozen.

## Data availability statement

The original contributions presented in the study are included in the article/Supplementary material, further inquiries can be directed to the corresponding author/s.

## Ethics statement

The studies involving human participants were reviewed and approved by Ethics Committee of School of Medicine of Xi'an Jiaotong University (approval number: 2020-1258). The patients/participants provided their written informed consent to participate in this study.

## Author contributions

SH and CC: conceptualization. CC: methodology and writing—original draft preparation. BY: software and formal analysis. CC and BY: validation. QL, CC, and BY: investigation. QL: resources, data curation, and supervision. CC and HH: writing—review and editing. BY and HH: visualization. SH: project administration. All authors have read and agreed to the published version of the manuscript.

## Conflict of interest

The authors declare that the research was conducted in the absence of any commercial or financial relationships that could be construed as a potential conflict of interest.

## Publisher's note

All claims expressed in this article are solely those of the authors and do not necessarily represent those of their affiliated organizations, or those of the publisher, the editors and the reviewers. Any product that may be evaluated in this article, or claim that may be made by its manufacturer, is not guaranteed or endorsed by the publisher.
