# Supplementary material for: Experience of rehabilitation management in public hospital after it was identified as designated rehabilitation hospital for COVID-19 patients: A qualitative study
Source: Front Public Health. 2022 Jul 26;10:919730. doi: 10.3389/fpubh.2022.919730 (PMC9362772; doi:10.3389/fpubh.2022.919730)
Supplement: Supplementary file 1 [file Data_Sheet_1.ZIP › Interview data/书记专访.docx]

H：说一下我们还有张静张老师还有参加。那个院长请你们先把医院目前情况给我们介绍一下。

J：行，那我大概介绍一下，大概说一下我们医院，这次作为新冠康复医院，按照专家组给我们的要求，我们腾出了整个一栋2014年新建成的住院楼作为康复病区。康复病区总共有12层，12层设置地总共床位是591张，再加上这个15张重症监护病床，总共是606张床。现在目前我们收治的康复患者是584名，也就是我们的床位基本上占满了。剩的几张床呢，因为有隔离病人，还有一些特殊病人要单间处理的，所以584就是我们的现在最大的容量。所以我们现在的康复病人呢，应该说是在全市里面是最多的。我们医院收治情况呢，从十号开始收治病人，截止昨天最后一个病人进来。相对来讲收治的病人都比较平稳，都是轻症的，不是太重。所以这个康复期呢，14天时间，14天以后要交给社区，再进行社区隔离，我们现在对康复病人的管理，医疗上的（管理），主要是一些简单的心肺康复的一些治疗，没有什么太特殊的，因为这个新冠肺炎，它本身的轻症引起了机体的一些气质性的变化不是很多。所以说就是一些简单的一些呼吸功能的锻炼，这个简单的一些治疗。再加上我们是综合医院，我们有一个比较好的条件，就是我们对这些病人的这个基础疾病的治疗我们还是能够跟得上。给我们的任务是除了新冠康复好以外，其他的基础疾病要保持稳定，不能出什么问题，所以我们的医疗保障主要是这两项任务。等会儿，如果我们的乔主任有时间的话，把医疗的情况给大家再大概介绍一下。然后我们主要的任务是保证医疗，还有一个就是防止院感发生。嗯，最近这段时间呢，西安市的三家定点救治医院。西安市胸科医院、西安市人民医院和长安区医院都发生了院内感染的事件。其中有50多名医护发生感染，然后还有再加上其他在医院感染的人员有160多（名），将近170名。所以这次医院院感的发生率应该说是这两年在全国是最高的，所以我们现在的还有一个重要的任务就是要防止院感的发生。在这方面，我们也下了很大的功夫，想了很多的办法，比如说我们现在从大的流程上我们进行改造，我们的入院病人和我们的工作人员分区域行走，病人和工作人员不在一个区域，不见面，不交叉。然后进了住院大楼以后呢，我们的大楼也是按照区域划分，“三区两通道”。其实我们的一个特点就是，整个把过去在一层楼里面做的感染病房，（通过）三区两通道的划分，我们把它立体化了，就成一个整个立体型的三区两通道的划分。也就说这整个住院楼经过我们这样一改造以后能够满足传染病房的最基本的院感的要求，这是一个。再还有就是我们对医护人员的院感培训。院感质的培训，特别是穿脱隔离服、防护服的这个培训。因为从其他几个医院发生院感反馈的情况来看，大部分（医护）感染可能和穿脱隔离服有关系。所以说，我们这个呢也是我们一个关键，我们对进入大楼的人都要进行穿脱隔离服的培训，特别是脱隔离服，脱隔离服是最容易感染的，因为他已经在病区里头工作过以后，隔离服上或多或少可能会带上一些病毒。如果脱隔离服脱得方法不对、不正确的话，就容易引起自己的感染，所以这个环节是非常关键。我们在这个环节上，在脱隔离服的环节上，专门安排了四个人在监督我们的人员，来把隔离服来脱好，这是一个环节。再还有一个预防院感的环节就是消杀问题，进入大楼的每个人员都有消杀任务，特别是我们的护理人员非常辛苦。不光是要看护病人，做基础的护理和护理工作，还要进行消杀。我们重点部位的消杀工作都是我们护理人员来完成。还有就是我们院内的其他区域的消杀，我们也都有安排，这是我们要做的工作。还有个就是我们每天要进行环境的监测。这从这十多天的运行来看，我们的环境监测都是阴性，应该说我们目前来看还是比较平稳的。环境如果不被感染，可能我们被感染机会就很少了，这是一个。再还有就是我们医院对在院的工作人员的核算检测。全员核算检测每天进行一次，这个也是刚才我讲的，我们王培书记负责这个医护人员管理组，他们来做这项工作，所以这个我们全员检测，我们连续十多天都是阴性，这个也是非常关键的。再还有就是我们人员的闭环管理，特别是住院大楼里面进入住院大楼的医生、护士和服务人员的闭环管理。这个也严格按照要求，除了在医院工作以外，（医院）每天工作六小时，其他时间全部在宾馆进行闭环管理，在宾馆里也是单人单间，不允许相互串门，不允许聚集。这个我们也严格按照管理，可能对我们防止院感发生都会有一定的意义。这是我们主要做的两项重要的工作，一项就是这个医疗救治，还一项就是院感防护。这是两个重要工作，还有其他的工作，主要是我们的一些辅助服务工作。我们这次所面临的一个非常重要的工作就是我们的保障和服务，也就是我们夏总负责的这一块，这个任务对我们来讲确实是一次考验。我们过去的服务保障，对病人的服务保障工作，除了医疗以外没有什么太多的生活上的服务，这次不一样，这次所有的病人生活上的服务，都需要我们的医护、我们的医院的工作人员来完成。比如说。这些病人在医院住的时间长，他的基本的生活物品没有，我们都得给他配送，当然，他是要掏钱的，掏钱要买的话，我们还得给他买回来，这个非常复杂，非常麻烦。还有个餐饮的问题，每天一日三餐，必须要保证一定的营养，饭还要可口，送到病人的门口，所以这个餐饮的保证也是我们所面临的，和过去不一样。我们过去的餐饮都是病人到食堂来买饭。然后拿回去自己吃。这次全部是我们的工作人员送上去，这个量也是非常大的，也是非常大。还有就是我们的其他的工作，保洁、保安这些工作都是和过去都有很大的区别，所以大家都是非常辛苦的，那这块呢，就是后勤保障这块也是做了大量的工作。然后还有我们的资金，从目前来看啊，这个也是我们面临最困难的问题，也是给政府提出申请的。我们现在所有的给患者的救助资金，包括他的餐饮资金，全部是垫付的，不用花一分钱，治疗不收钱，这个吃饭不收钱，他的一些基本的一些生活物资我们也是免费的，除了有些特殊需求以外啊，这些东西我们都是免费的，资金压力也是非常大，我们夏总在这方面也是做了大量的工作来筹措这个资金。那这块呢对我们来讲也是非常关键的一个，如果说我们给患者服务不好，患者的投诉就会很多，因为现在目前我们也接到一些投诉，个性化的投诉也是非常多的，但是我们个性化解决也还好。还有个就是，我们是医疗机构，我们不会像宾馆酒店那样给他们提供那么好的服务，我们也通过一些方式跟患者进行沟通，沟通好以后取得患者的谅解和理解。最近这一段时间呢，经过这么快七天的这个磨合，已经和患者达成了非常好的，可以说非常好的一致吧，能理解我们医院现在所面临的问题和我们所能提供的服务，这个是我们在做的一项工作。

医院里面还有几项工作也是比较困难的，比如说患者的转接问题，从救治医院、定点医院转来的患者要平稳地接到我们医院病区，这个对接工作也是非常复杂的。这块呢，我们李小凤李主任做了大量工作。我们最多的一天接了150个病人，150多个病人。我们在接病人过程中，由于我们前期准备比较充分，沟通比较顺畅，病人到我们医院以后，没有出现一例在医院内滞留的、没有安排好的病人。在这块呢，我们也想了很多办法，下一步可能还有病人出院的问题。病人出院的，除了在我们医院要做好登记以外，还要和社区进行沟通。为了把这项工作做好呢，我们又成立了这个出院和社区对接的一个工作小组，也安排了五六个人来做这项工作，也是为了保证患者能够顺顺利利的从我们院出去，和社区进行交接，保证患者在这段时间无缝衔接，这块工作也是大量的工作啊。还有就是我们护理上，现在不光是要做一些护理服务，还有一些人员管理上、院感的监督。因为我们医院这个院感科人员相对来说比较少，四个人，有一位被抽到市人民医院做支援了，现只有三个人，我们就发动了我们所有的护士长和我们的感控员作为我们的监督员，来加强我们的院感监督，目的也是为了防止院感发生。这块任务也是非常重的，特别是我们护理上有一个转型。叫什么转型，我们除了打针发药这个工作以外，我们还打扫卫生，转型成为一个保洁员的这个工作，所以对我们护理人员来讲也是一个考验，但是从目前来看，做的工作还做的还是比较好的。这是我们院内的一些工作，还有一些对外的工作，也是非常的复杂。我们张小刚助理负责我们整个的对外协调工作，我们对外协调主要一个是和政府间的协调。西安市这个疫情防控指挥部给我们下派的任务，我们要对接起来，对接起来以后我们又要完成怎么样和政府对接；还有个对接就是要和我们的宾馆酒店对接。这个宾馆酒店都是政府指令性的一些酒店、宾馆，管理起来也是非常困难的。所以，这种对接工作也是非常非常的重。我们现在外面有四个酒店，住了我们的人。每个酒店都要做好相关的管理工作，要防止在酒店内发生院感，所以这块的任务也是非常重的。还有协调其他的社会上，比如我们的社区、我们的办事处还有我们的卫健委、卫健局很多的协调工作，我们小刚助理也是有大量的工作。这种和政府之间的沟通啊是非常关键的，我们这次能够接受这任务，不是说我们主动要接受这任务，也是说政府对西安市所有的医院都进行过考察。一个是看上我们这个环境，相对来讲这栋住院大楼能够满足相关的院感的要求，作为一个康复期的患者救治的一个要求。还有最重要的一个原因就是我们和政府之间沟通相对比较平和，容易沟通，相互能够理解，理解以后我们的工作，政府的工作就好安排好布置。所以说可能也是我们好说话吧，所以这个任务就交给我们了，所以这块呢。我们也想通过这次的工作呢，我们把和政府之间的关系建立得更加紧密。最近西安市省政府、省卫健委还有新城区政府、卫健局的领导，对我们都是非常的关心。刘金社书记，昨天下午2:30打电话，慰问我们的职工，今天中午，杨林张副主任1:30到我们医院来检查，也是来做一个慰问，也强调了我们院感的问题，也给我们解决了很多我们现在面临这些困难。今天下午新城区政协主席、新城区副区长、新城区卫健局局、还在办事处的书记，还有我们这块幸福林带改造委员会，他们的领导都到我们医院来，对我们医院来进行慰问。所以说我们通过这次的转型，成为康复医院，应该说把我们和政府之间的关系拉得更强。特别是方省长对我们医院接受这个任务，而且在短时间内能够完成好这项任务，也给了非常高的评价和肯定。所以对我们来讲都是一个非常积极的信号和鼓励啊。所以我们最近在工作呢。我想给我们职工应该说是一个非常大的鼓舞，特别是领导的关心。昨天下午我们开中层的视频会的时候，我把刘金社书记对我们职工的一些问候和关怀也传达给我们的中层干部，也给我们的职工也有很大的信心，所以说，我们也是想通过我们自己的努力，把这次的这个任务完成好。

胡教授，我把我们医院的最近做的一些基本的工作再介绍一下。对了，我们还有两项工作在做。就是除了我们这个新冠疫情康复医院的一些基本的工作以外呢，我们还有一个就互联网院的工作也在做。现在我们医院目前是一个全面停诊的状态，我们作为一个综合医院我们的业务。现在可以说正常的业务都关闭了，停止了，但是我们不想把我们的业务丢掉，不想把我们的患者失去，所以我们通过互联网诊疗这种方式呢继续为我们的患者进行服务。这个服务呢，应该说是有一定的效果的，能够把我们的一些老的患者能够吸引到我们的互联网医院上进行一些在线咨询和一些疾病指导。这项工作我们也在做，因为互联网医院是我们刚刚自己在建，我们有自主知识产权的一个互联网医院，在这块呢，这互联网医院我们也确实在这段时间发挥了一定的作用。我们下一步呢，还将通过互联网医院，除了开通咨询业务以外，我们线上的服务要更加扩大，比如说我们的送药的问题，还有我们现在护理部开展的护理延伸服务，居家的这个护理问题都可以通过线上的这个预约来完成。所以这块工作呢，我们也是在抓紧时间来做。这是我们最近在做的一些工作，我想给胡教授和您的团队汇报就这么多。
