# Supplementary material for: Experience of rehabilitation management in public hospital after it was identified as designated rehabilitation hospital for COVID-19 patients: A qualitative study
Source: Front Public Health. 2022 Jul 26;10:919730. doi: 10.3389/fpubh.2022.919730 (PMC9362772; doi:10.3389/fpubh.2022.919730)
Supplement: Supplementary file 1 [file Data_Sheet_1.ZIP › Interview data/事业发展部主任-负责对外协调和互联网项目.docx]

H（郝主任）：教授好！然后我从我科室的这个职能来谈一下我对这次我们医院作为新冠康复定点医院的几个想法和感受吧。那么我是在事业发展部，其中这个科室，它有一项职能就是协助医院的战略规划落地实施，同时互联网医院的这个管理项目也在我这个部门。在这一次这个新冠的抗疫当中，我也主要参与的其实也就是两项工作。第一个项工作就是对外核酸采集队的工作，第二个就是互联网医院的工作。那么在谈这个核酸采集的工作，我只提一个小故事，分享给教授以及教授的团队，还有我们在座的院领导，还有咱们科主任们听一下。从初始阶段，我作为队长来说，我们这个团队一共有100人这样的核酸检测队，然后大大小小也出了很多趟任务，其中有一趟任务的就是让我记忆犹新。是被抽到一个高风险区雁塔区，然后我们是下午五点走的，但是我们回来之后是第二天凌晨的五点，也就是在这种长时间的高压的这种作战状态底下，当时发生了一件事情，就是所有的人都在十二点的时候已经把自己的工作做完了，而且在做的过程当中，都是分了八个小组过去，每个小组都是尽快的把自己的工作完成了，赶紧给我打电话，又拖到另外一个场子上去给其他的队员去支援，这样的话能保证我们的队伍能早点回家。当把所有的这些都采集完了之后，我们接到一个电话，就是当地的一个丈八路社区的，当时说，他们还有两个队员回不来，希望我们能支援，但是那个时候我们所有的人都已经上车了，而且能看到大家都已经非常疲惫了。整个楼上去之后，打开一个黄码，打开一个红码，都是这样的一种风险状态下，然后我当时就在车上问了一句，我说我准备过去支援一下丈八路的同志，咱们车上还有精力的，你们跟着我下来，实在身体也不舒服的、累的就不要下来了。我前脚一走，我后脚转身的时候，我才发现车上的所有队员都下来了。（丈八路）那儿还不是一批人，当我们进去之后，对那个区域里面是两眼一波黑，不知道里面还有多少任务量。但是我进去了之后，我们的队员就发信息说队长我们进来支援，我告诉他们说不要，因为越多的人进入到这个未知的环境当中，管理风险会大大的增加，我不敢保证我的队员在这个环境底下会发生什么样的一个事情。但是，当我们每下一层楼时候，我就发现我一个小组到了，在下一层楼的时候我另外一个小组又到了。最后导致这一栋楼，一个小区里边全是我们的队员，然后完了之后，我们在另外一栋楼看到了两个队员不是我们的人，在电梯里边两个蜷缩着，是太累了。然后当时我们所有人的眼眶都湿了，其实我讲到这时候，我其实还是心里有点激动的，说这个的话是想说我们的这个团队在初建的100人，到后边被减成88，减成68到减36，但是工作任务一点都没有减，不仅是说我们彼此之间的协作能力、团体作战能力增加了，我们的经验和技巧增加了，然后更是对我们整体这个医院的统一的协调能力，还有后勤的保障能力，都是一种考验和进步。也就是说，教授，其实您听完我们前面的院领导还有科主任说，他们大多都说到了非常暖、非常感人的，还有我们的团队的协作方面、沟通能力方面其实已经感受到了，就是我们整个四院在这次面对新冠康复定点医院的设定，我们所面临的机遇和挑战下面，我们医院成长了，我们团体作战能力和奉献精神体现出来了。而且从我们后续的这块管理上来说的话，给我们未来的文化建设奠定了一个非常扎实的基础，也就是说，我们医院的核心的文化产生了，内部协作的机制已经初步形成了，这是我想说这次新冠疫情和我们作为定点康复医院来说，对于我们这个医院和我们这个大的家庭，我们这个团队来说，给我们带来的这个机遇，这是第一点。

还有第二点的话，互联网医院的建设项目和运营，也是在我们部门放着。那么，关于医院目前的这个运行的状态来说的话，我们院领导，夏总，这块都已经说了，我们现在每前行一步都是非常非常的难，所以我这块考虑的话更多的是在复工复产上去着手，也就是说，当我们把这个新冠疫情的最后一个康复病人送出医院之后，我们医院如何能在短时间内快速的恢复生产并且运转起来，我觉得这个互联网医院建设项目应该是可以有一定的促进作用，所以我提几个想法，也请咱们胡教授还有我们的院领导还有科主任们帮我把把脉，看一下我的这个想法，成熟不成熟。那就是说，结合咱们这次新冠疫情期间我们作为新冠的地点康复医院和医院未来转型健康管理型医院的这个发展定位的话，结合目前我们这个部门还在做一项国家的项目，即“5G+健康管理”试点项目的实施，那么要充分地发挥“5G互联网医院”还有物联网技术的优势，还有我们目前作为区域的城市的这个医疗集团，三级加基层的体系化服务的优势，那么我想的就是在互联网的这个平台上开展一些新冠患者、出院患者的“互联网+健康管理”的这个服务。那么也就是说除过做好社会任务之外，我们怎么样能把所有在我们医院出院后的患者能进一步深入地去管理，不只说把他这14天管好，把他14天出去之后再管好，让我们整个省四院的这个影响力能更大一步地去延伸和扩张起来。那么第一点，我打算在互联网这块成立一个在线的康复和健康管理专班。在互联网医院这个平台上去开通互联网新冠肺炎患者的在线健康管理服务。那么这个的话，教授您在微信小程序上向下拉，搜“陕西省第四人民医院”就能看到我们医院目前这个1.0版本的互联网院建设。那么目前我们在线上开通了图文咨询，在开通的将近十几天当中，我们现在已经接近2000多人的服务量。我们后期的打算就在这个板块里面再开一个在线的新冠患者院后的管理服务项目的板块，通过这个板块呢，也就是第二点，开通居家健康的监测服务，提醒患者定期使用5G居家健康监测设备，测量和监测他的体温、心率、呼吸、血压这些数据，定期进行上报，而且设置一个预警，预警系统会提醒我们这个专班人员，也就说提醒我们的护理团队及时的对患者进行确认、干预和处置。这样的话就是让患者觉得虽然说离开了我们医院，但是他的服务我们依然是通过互联网延伸到他那边。第三个的话，我还希望打算通过开展一些健康的康复训练活动。他在病区主要是我们医护团队在帮他去做一些康复的服务，但是他回家之后，我们就会找一些团队，我们医护人员把这些编成一些视频放在我们的互联网医院上，他可以每天跟着去看。还有就是开设一些直播课程，让我们的医护团队在线上，然后定时的给这些出院的患者做一些健康运动的打卡，增强他们的免疫力，提升这个康复质量。第四点就是我们目前依然在做的这个在线的心理咨询服务，同时邀请一些其他医院的心理专家入驻我们的互联网医院，为康复患者提供及时的一个心理辅导和干预。第五点的话就是开展在线的营养服务，开通营养咨询，然后邀请营养专家拍摄一些有针对性的科普式的视频。指导患者在居家的时候，保证他的营养健康，为他们康复打好一定的基础。第六点就是跟踪随访，这块初步打算是按三个月、六个月、十二个月进行随访，手机端会自动的生成一个随访页面，患者填写随访记录，对特定的人群，比如说老年人，还有一些基础性疾病的人进行标注，这样可以有针对性的给予一些干预。那么第七点的话就是开展一些问诊、复诊开方还有一些“互联网+诊疗”的一些其他服务，为居家的患者在康复期间提供些医疗和药品的配送服务，然后满足患者其他疾病的医疗方面的需求。教授、院领导我就汇报到这。
