# Supplementary material for: Experience of rehabilitation management in public hospital after it was identified as designated rehabilitation hospital for COVID-19 patients: A qualitative study
Source: Front Public Health. 2022 Jul 26;10:919730. doi: 10.3389/fpubh.2022.919730 (PMC9362772; doi:10.3389/fpubh.2022.919730)
Supplement: Supplementary file 1 [file Data_Sheet_1.ZIP › Interview data/助理-负责院内康复救治和院外核酸采集.docx]

J：唉好，来看看我们其他同志有没有什么可以要补充的。张助理先来说一下。

Z（张助理）：胡教授你好！我谈一下整次这个在疫情防护过程中的一点体会吧。我觉得这可能还是管理上的问题，正好您在这块我们看到的话，我想这个时候，我体会非常深刻的一个直观感觉吧。因为我们实际上是承担两份工作，一份是院内的康复救治，还有一份我们参加了整个西安市新城区雁塔区高新区的一个核酸采集。截至昨天下午，我们医院承担了103万的这么一个核酸采集量任务，那么在本次这个核酸采集过程中，我们体会最多的是一个，就整个大环境嘛，我们感觉反正在这个我们有啥就直说就。一个是管理比较混乱，整个是一个管理上的问题，有时候我们直接感觉就是一个手忙脚乱，整个工作就是一个疲于应付，可以说是事倍功半。举个例子，原来的话我们要采2万人，要150个人，大家都在工作。昨天我们30个人，（采）2万人两个小时，不到三个小时全部干完了，干的效果还非常好，然后的话，这个居民也满意，政府也满意，我们干的也舒心。那么在这之前是不可想象的，大清早六点多把大家号过去以后，大量的工作，大量的对接，都在忙乱，但就是手里出不了活，所以是，最后这个好多工作我们感觉是平静一些，也没有效果，其实在这个采购过程可能也会造成潜在性的感染，有这种可能，这是个大环境。所以这次我们接的这个新冠康复定点医院这个工作以后，其实压力挺大的，因为在三个定点医院都发生院感事件。从国家卫健委给我们的指令是康复院绝对不能再出现这情况，但是在这过程的话，病人虽然从这家定点医院是正式出院的，符合出院的指标，但也有复阳的可能。所以，对我们来讲也是用了仅仅30天时间完成康复医院这么一个，整个不光是建筑布局上调整，整个管理思维、组织构架，还有我们所有人的这个工作职责和职能（的调整）。刚才院长提到的，现在不是看病，整个是一个管人，包括对我们的这个保障能力都是一个很大的一个考验。因为病人住到医院以后，原来主要是看病，现在就是要生活，要调节心理，外面的快递不能往里送。病人经过前段时间这个治疗以后，在定点治疗医院他觉得是个病人，病人的依从性还是比较好，到我们这以后，他就觉得自己出院了，他的需求马上就发生变化了，所以一些负面情绪这两天就是暴露比较多。所以这对于我们接到的投诉也是比较多，都在继续进行处理。所以说是基于这个大环境以后，我们这次接到这个任务以后，其实我们还是把重点放在这个管理上，管理的话首先就是从管理构架、工作流程、工作职责、督办落实这方面我们是提早着手，而且在整个救治过程中（医院）一直在这方面加强。我们的工作手册一直在改版，每天都在新的上补充，那么想这次可能是一个很好的经验积累。总体来讲，目前就是我们是边干边总结，边总结边提升，所以我们相信有了一个科学的管理的话，我们这个康复院这个工作就说是会有序进行。其实的在三所定点院发生院感的事件，这个也不奇怪，其实医学措施从武汉已经有了一些经验，标准是一样的，那关键就是在这执行过程，组织管理造成的这些不必要的（问题），出现一些混乱引起的不必要的感染。你像昨天我们听的数字，50位医务人员被感染，1700多位医务人员被隔离。正是用人之际，造成了这么一个无谓的牺牲，我觉得非常惨重的一个教训，所以说在我们这一块的话，我们把管理还是放在最前面。管理做好了，我想所有的一些措施落实到位，那么这个工作的话，不管这个疫情在持续多长时间，但是我们的工作应该不会出啥大的问题，我就补充这一点。
