# Supplementary material for: Experience of rehabilitation management in public hospital after it was identified as designated rehabilitation hospital for COVID-19 patients: A qualitative study
Source: Front Public Health. 2022 Jul 26;10:919730. doi: 10.3389/fpubh.2022.919730 (PMC9362772; doi:10.3389/fpubh.2022.919730)
Supplement: Supplementary file 1 [file Data_Sheet_1.ZIP › Interview data/医务科主任-兼绩效考核办.docx]

L（李主任）：我大概很简短。我因为负责了一个很细的一个细节，我是负责患者入院的这个流程，我对外对接的是我们西安市疫情指挥部、西安市的急救中心，还有三家的定点医院，在这中间的一些细节，因为我接到这个任务是提前两天接到。然后首先第一点把我的这个思路先理了一下，做了一个流程，还有我们患者交接支持的东西，然后在这个对接的过程中一些细节我就不说了，就是我谈一下在这个事情过程中，我觉得还有可能从我们，当然这个改进可能不是医院这个层面去能改进的。第一个就是整个信息交流。一个患者要送一把钥匙进我们病房，我是用两个半小时去解决这样的一个问题。就刚才我在去开会的时候，我们要来一个病人，这个病人应该是在五点开始跟我们讲，打电话说要来一个病人。刚才大概就是七点，不到7:30的样子，病人来了。就是一个事情，我们要沟通，沟通的成本很大，因为他在沟通的时候没有模板化，没有知识化。导致信息的整个（流程）就会比较乱，所以我觉得这一块我们三家定点医院，从政府层面上来说，对这三家医院在定的时候没有立规矩。就很简单一个事情，我举个例子，当时我们想着这么大批量进来，我就设了一个表格，就是你提前前一天，你明天要出院的。你今天你整个的信息系统，你从你住院信息系统HIS里面导出来，包括患者的基本信息，然后你交给我的时候，我按照这个基本信息，我会提前给你把这个手续办好，然后我列了一个这样的表格。然后我给我们三家医院去对接的时候，没有一家医院用我的表，因为啥呢，他们说你去跟秦皇去取经验，秦皇怎么去办了，为什么你们就办不成？我当时的这个理解，最后我跟西安市卫生局去说。卫生局说是，那还有一些表格是啥，有用word版的，有用excel表的，我就说老师能不能我们统一用一个ecxel表，我们后期好统计啊。老师说，那你自己不会把word导成excel表，我说我导了，我是为了我统计方便，我担心你后期统计的时候不方便，然后就再没有回信。就从这一点来说，在我们信息化建设，还有我们这个交流的时候，从顶层设计来说。没有去有一个很规范的东西，所以就是仅仅是满足于把这个事情完成了。然后导致后期是啥，西安市政府他也是西安市疫情指挥部。他来跟我要我们医院在院病人的数字，我说这个不是西安市卫健局能给你提供更完整的信息。我这天天都是在动，因为啥呢，天天都是有入有出，虽然说这两天没有大批量的入和出，但是每天都会有入都会有出。这是我觉得在这个搭建的时候缺少的。第二个就是我们要建立一种形式，就是从我们去年新冠开始的时候，政府一直在倡导说，建立一种平战结合的医院的院区、多区的医院管理模式，就提到很多，但是我觉得平战结合在陕西这一块，平战结合好像从医院整个资源的调配上来说，还是比较欠缺，包括我们硬件的一些设施，还有我们人员的调配。就举一个很简单的例子，我们流调人员的缺口，流调人员缺口就是我们病人最多的时候，一天发生几十例几百例的时候，我们疾控中心的人员肯定是不够的，就是在这样的情况下，我们政府没有怎么想发挥我们各家医院，因为各家医院都有预防保健科或者疾病防科，这样的人员就是我们流掉队伍的后备队员。然后在医院里面，怎么样再去组织他们这样的一个体系，也就是流调队伍的一个系统化建设，从政府层面上，我觉得在这一块可能需要做一些。当然，从我刚开始接这个工作来说，因为就是病人的一个住院，我跟他们对接完，我说叫周帅群发消息，我们今天大概入几个患者，然后我们后勤保障部门根据这个去配物资，然后我要去保卫科通知，几点钟第一辆车来，你要按时给我打开门，然后我要通知我们转运组这个病人第一批的车次来，来几个病人，然后他们人员要到位。然后我们医疗组还会在这个中间提前会给我去办好住院手续。所以各个部门配合来说，我觉得我们院内是很团结很顺，没有在我们医院发生医院车、病人的堆积。我们要求就是如果来两辆车，第一辆车的人在下的时候，第二辆车绝对不能动，不能下人，人一下车就乱了，车好管，因为车里面他拉了四五个人，就整个很顺而且我们没有积压。院内我觉得在这次整个我们抗击疫情的过程中，各个部门就像夏总说的时候，我说呀，院长真的是咬着牙在往前挺，一个月几千万的这个资金垫支，这个确实压力很大。还有一个就是从我们院内的病区的管理，这也是我自己可考虑的，因为就是前两天我总值班的时候，当然我没去值，是我们另外一个同事帮我值的。就是患者现在可能有20%甚至30%的精力去做治疗，还有大量的闲暇的时间我们怎么样去（给他安排）。我今天还在想我们有没有可能去给他做一个作息时间表，要让他每一个时间段都有事情干，这样的话我们对这个康复病区的管理形成一个模式化。比方说，早上7:30或8:30，这是你起床洗漱时间、个人卫生，还有你房间卫生的打扫时间，或者几点钟是吃早餐时间，然后是我们医生查房时间，然后下来是你做操和锻炼的时间，我们有没有可能去做，就是让他从早上起床到晚上休息的时候这样一个模式化的东西。然后当然有个性化需求的时候，可以不去按照（时间表）。比方说，我今天突然血压高，我要去会诊，那这样是特殊的例子，然后患者他也要知道在我们医院的常规性东西，比方说我要在第12天第13天我要查什么，第14天我要查什么，查完以后，下午就是我的志愿时间。患者对这些情况他不知道，他现在目前做很多东西还不是很清楚，导致他现在觉得我们的饭不好啦，我们护士、医生查房的时间短了，就各种各样的事情，我觉得没有让他忙起来，这有没有可能去做一种知识化的作息时间表，让他有事干，甚至有些事情可以让他去参与一下。因为他现在是一个心身的一个康复，不是生理的一个疾病的康复，我觉得这一块可能对于我们康复医院还能去做一点点事情。当然，最后一个就是关于他们出院以后我们怎么样去做他下一步的一些健康的指导。可能我们要根据患者与我们医院周围的这个距离去做一下我们有针对性的，因为毕竟医院这些病人，政府也是很关注，因为他要定期还要到原来的就是定点医院去复查。作为我们来说，让他能进到我们医院一次，怎么样把方圆三公里还是五公里这个患者，他来了，他以后有病了，在我们正常开展的时候，也会去认可我们医院，所以这可能是我们下来要考虑做的一些事情。我的发言就完了。

谢谢教授。
