# Supplementary material for: Experience of rehabilitation management in public hospital after it was identified as designated rehabilitation hospital for COVID-19 patients: A qualitative study
Source: Front Public Health. 2022 Jul 26;10:919730. doi: 10.3389/fpubh.2022.919730 (PMC9362772; doi:10.3389/fpubh.2022.919730)
Supplement: Supplementary file 1 [file Data_Sheet_1.ZIP › Interview data/大内科-负责护理团队管理和院感防控.docx]

Y（余护士长）：那我说一点啊，因为我这也就是给我们护士发个声啊。胡教授，您好！我作为我们护理部就说大内科的护士长，我们主任今天也没过来参这个会。我代表我们全院的一个护理队伍，在这也就发个言。我们医院也是作为西安市定点康复医院，也收治了我们第一批的584名康复患者。在这康复期内，我们的这些患者可能在住院期间康复了，（到我们医院后）他以前的那些需求可能就爆发式的一个增长，弄的我们保障这边压力也大。我们其实在病区的一些护士的工作压力也是蛮大；另外呢，我在这也想说，我们护理队伍在刚开始在组建要进这个病区的时候，也是我们这个康复病区组建的主力军。我们康复病房的医护人员是分两批次进住的，一批可能工作14天，那么我们这两批的总共的医务人员是557名，我们的护士就是303名，包括我们医院前期的可能在核酸检测这方面的工作，其实作为护理这也是绝对的一个主力军，目前来说就是我们康复医院对患者的这个服务，目前是我们最主要的一个工作任务。那么对我们这个患者在入住前，我们护理也做了大量的工作，比如说，我们也确实和秦皇医院进行了交流，我们也问了人家这些病人的要管理的一个模式。然后我们也制定了我们医院的针对这个患者的一些护理常规、护理规定、排了班，也做了一些职责，那么在病人入住以后，我们也做了我们的一个入院的须知。给患者在治疗过程中，我们从护理这儿，因为这个患者他的康复期可能治疗确实比较少，就是基础病的一些治疗（比较少），那么最主要的可能还是一些康复，这儿就是护士会指导他们，做那个俯卧位的一个呼吸，这个就是说，可能这个患者从定点医院出院，他也养成习惯了每天两次俯卧位的呼吸，一次就是30分钟左右，那么还有每天两次到三次的一个康复操，就是我们做的就是呼吸操的一个功能训练，每次也就是大概就是15分钟。还有呢，就说从康复这，我们可能就是做了这么多这些工作，那么下来就说是，在这个保洁方面。因为刚才我们院长也说了护士可能不单是要给患者去指导他的一个康复，那么还有一些保洁的任务。在病区可能由患者去进行一个基本的清洁，给他发一些消毒湿巾，在公共区的卫生是由我们护士去做的，每天至少有一次公共区卫生的打扫。另外呢，像物表，像门把手这些每天都是（清洁），必要时我们都不停的再去做这些事情。还有对患者，我们在接触这十几天以来，其实感受最大的就是他那个需求比较多，然后可能沟通起来就比较困难，我们也接到了一些投诉，不管他是说你护士没有按时给他消毒，或者是找了一些没有按时的满足他的一些需要，但是我们通过分析以后，我们认为这个患者可能还是比较压抑，可能关的时间比较长，所以有这个焦虑、紧张、抑郁的这种情绪。所以从这些方面呢，我也是给我们的护士长也在说，我们一定要跟患者要加强沟通，去了解患者的需要，有时候可能我们确实也解决不了他的一些问问题，但是只要他把那些话说出来了，把他的那些不满发泄出来了，可能对他的这个情绪也是一个疏导。那么我们也想了很多办法，比如说是我们有些病区可以建一个医护患群，患者可以在群里头去发表一些心声。刚开始，可能牢骚特别多，大家建的时候可能觉得我们这个群是不是建错了，你看负面情绪这么大，但是运行了几天以后，现在就逐渐的就平静了，大家好像就理解我们，都互相在这沟通，允许患者去发泄这种不良的情绪。那么而且这位护长也跟我也说了，今天给我发的信息，说是她今天去上班的时候他们有一个患者就说“哎呀昨天我没有看到你来，我心里还挺想你的”，然后她觉得她的一些努力，其实患者的需求可能就是他说完以后，我们满足他了，他发泄完以后，他可能也就认可我们的工作。还有就是我们病区在每天给我们的患者做一些宣教，我们会有病区广播，每天做三次，而且我们会去督促患者去锻炼，因为病房确实空间有限，那么每次我们也发现患者这个运动量是严重的不足。基本上，我们每次去巡视的时候，他们都在床上看手机，或者睡觉，这个运动量也不足，运动量不够，心情也不好，这样的话对他的康复也不好，所以我们还是护士会去督促他们去做这个运动。在这我想跟胡教授提出我们的一些疑惑，就是我们的对心理方面，虽然我是很浅显的认识，我认为这个患者可能这个不良情绪是需要疏导和宣泄的，但是还是缺乏一些专业的指导。我们医院里心理科的医生也在做这方面的调研，还是希望我们能得到胡教授团队的一个更专业的指导。还有就是护士这儿她们的工作是非常卖力的，不管在病房、在核酸检测，还是在我们康复病区的护理工作中都是非常卖力的，包括除了我们刚才说的那几项工作以外，其实大家给我反应病房的水堵了，也是护士去通的，就是（病房的水）拔不开，然后拿铁子通也要把它通开。很多的工作反正保洁可能不好进去，然后我们的总务可能维修也不太好进去的时候，这些工作其实都是护士在干，他们是任劳任怨，其实他们患者是一句很简单的“哎呀，我都想你了”，他们就满满的感动。其实我在这就想说，我们的护士太需要我们的同行和患者的认可，谢谢，我就说这么多吧。
