# Supplementary material for: Experience of rehabilitation management in public hospital after it was identified as designated rehabilitation hospital for COVID-19 patients: A qualitative study
Source: Front Public Health. 2022 Jul 26;10:919730. doi: 10.3389/fpubh.2022.919730 (PMC9362772; doi:10.3389/fpubh.2022.919730)
Supplement: Supplementary file 1 [file Data_Sheet_1.ZIP › Interview data/总会计师-负责财务大权.docx]

X（夏会计）：胡教授好！我就是说一下，因为我这次物资保障啊，管人财务，但是我这次有两个深刻的体会。第一个就是这一次对这个物资保障的费用远远超过了我的想象，我不知道这个数字能不能说，我得请示一下院长

J：可以可以，我们也知道。

X（夏会计）：我跟您说按类别来说啊，我们这次的费用呢，牵扯两大块，第一呢，就是患者所有的医疗支出是我们院全部垫支。那么新冠康复患者实际上的费用并不多，就是说他发生的救治时的费用很少，但是平均下来的一个人就是14天就住（下来），每个人能收他们1600多块钱，是很少。那么这个费用呢，尽管是每个人的费用只有1600多块钱，也是由医院垫支。首先，我想说的是，新冠患者入住我们康复医院以后呢，他的医疗救治费用是我们医院垫支的，这是一大块，第一个，这是最小的费用，一个月下来就是166万。第二款主要就是对于康复患者的保障费用，里面分了三个内容，第一块呢，就是闭环管理费用，什么叫闭环管理费用，就是所有的康复患者，我们住了585个人都在我们的住院大楼，你也见过，在住院大楼里面的闭环费用，首先我们说一下，进入这个病房585个人的12个病区里面，我们进入了300名医护人员，但是为了300名医护人员，我们楼外面还有180名保障人员，保洁、保卫，还有这些其他的这些保障人员。那么这些人员呢都是闭环的，是要医院提供住宿的，那么光他们这个住宿费每个月994万元，我们租了四个酒店，还有医院，在医院的这个剩下前面门诊楼里面还住了好多（保障人员），（如）这些保洁员都不能回家，餐饮的做饭的这些工作人员不能回家，餐饮服务员不能回家。还有我们提供的这个车辆费用每个月就是18万，就是我们所有的救护车，来运运转病人，还有包括我们把这些工作人员运到酒店去、接来酒店来、闭环管理的早餐，这是第一大块就这么大。第二个就是我们的职工的费用，我们医院有1100多名的职工，现在分了两部分，一部分用于这个参与新冠疫情的工作，第二部分，人员好几百人，300人是参与核酸检测。还有部分行政人员，还有部分是居家隔离的，那么居家隔离可能占到我们1100人（中）也就是一二百人的样子。所以我们2/3的人投入到新冠疫情的救治和康复的工作当中，但是我们的人员费用一个月下来就是880万，这里的工资是660万，我们的社保是220万，所有的人员经费880万，也是我们院垫支的，政府没有投一分钱，我们要给职工还要发工资。这里面就根本没有包含我们现在投入疫情防控应该给大家的补贴，很多人是24小时转，很多人是核酸检测，那个您也知道穿着防护服在外面一干一个昼夜的，披星戴月地在干，目前没有一分钱的补贴，大家都在为这个抗疫在做贡献，这是我们最大一块。还有就是防疫物资，您也知道，这个新冠的康复主要是要来自于防护，我们要做到零感染，医院的防护物资投入是非常大的，我们现在一天N95口罩都要消费1000个，所有的隔离服，一个隔离服就是50块钱，这样算下我们一个月的光防护物资基本就是150万。这样算下来，以上几个费用算下来之后，我们一个月现在的投入就是2498.6万，这个数字很让人吓一跳，也就是毛2500万。这里面还没有算我们医院日常的水、电暖的运行费用，（这些费用）一个月是150万。这里面我们都算是基本工资，按6000块钱人均工资算的，还没有含奖金。这样您想，我们所有的这些支出全部是医院垫支，我认为在新冠疫情救治工作当中，各级政府都到医院来关心了，但是带来的都是慰问和关心的，我们的资金没有到位，作为我管什么喊什么，那我觉得资金一分钱没有到位，这个政府是应该给我们拨付一些资金的。我觉得政府首先要投入我们，我们投入这个工作就应该有（资金），就像我们立项一样，科研立项还有一个投入资金，我们作为康复医院替政府承担这个工作，不说政府买单了，至少要有我们的启动资金、投入资金、买单资金，这个我认为还是不到位的，我们现在就是说全部由医院垫支的话，我就给您算个大账，2500万我们账面上就是个8000万，2500万，三个月就是7500万，西安市清零就是我们医院清零最后一个病人，标志着西安抗击疫情战斗的结束。我认为西安现在总共2000多个患者，新冠患者最终要从康复医院出院，我们院是承担康复医院的大头。我想着，两三个月，最少两个月吧，三个月7500万，如果政府再不投钱，我们账面就剩下两三千了。我们医院不承担新冠疫情，正常一个月运转，必须有2000多万，所以这个资金压力是非常大，资金缺口也非常大。所以我也就是在最短的时间，我们也去给贾院长呼吁啊，真的是极大的呼吁，我们也能够看胡教声能不能通过学术界，也能够体谅到我们医院的困难和替政府承担的这种态度吧，我就可能补充这个说的多一些。另外我想在物资保障上我给您说几个我们如何开展这工作，我觉得其实我们院这次的承担这个新冠康复医院体现了很多管理上的这种高效的运转职能，我们医院分了八个专案组，我其中负责的是保障组，我的保障组有五个职能部门——财务、总务、信息、医疗设备和药学部，还有我们的医学装备部六个部门，承担了六大块的内容。您像第一块财务，首先要承担的是资金保障。我们在这里面设定了两个流程，一个是新冠患者来了之后，我们医院所要建立两套财务信息系统，过去我们叫内科，外科，现在我们把病区叫新冠康复一、康复二。首先，我们在系统的设置里面把系统赶紧做了个更新啊，新增加了我们新进病区的660年的医护患者的个人信息。

这样我们病人进来之后，跟我们原有的省四院的结算系统分为两个模块，这是我们信息化的建立，结算上首先要分离开。保障我们问政府要钱，我们自己算账要能够算得清楚。第二块就是我们的后勤保障组，后勤保障组除了我们的这个队服电梯物资以外呢，我们还设了个餐饮组。你也知道，585个人的三餐，一日三餐，这个饭我们要保证饭不凉，我们要保证585份在40分钟内送到，大家这个压力也是很大的，还要承担我们不被感染这样一个风险，所以我们医院院长及时设定那个配送组和转运组。也就是，我们配餐的人是在楼外配餐，再转送到楼内。楼内的这些接餐的人员他们叫转运组，他们接到我们从外面推送的物资以后，他们是全部的隔离的这种装置，他们再进行配送，保证了我们的餐不凉，还能快速传递到患者的手里。第二个我们在做餐饮的时候做了个特色服务，我把所有的餐设了几种，一个是宝宝餐，我们最小（患者）只有11个月，宝宝餐、儿童餐，还有糖尿病餐，这样的话我们在这种餐饮服务上，还有糖尿病。接下来我们将跟护理团队一块做，给患者过生日，提供温馨的服务，这是我们的餐饮。那么在药品和医学装备，我们这个方面也是用的是导班的形式去给他们送药，特别在医学装备的时候，我们我们要减少人员流动和进入这个楼的人员。所以我们给整个12层的病区设置时，在第三层楼的时候，设了一个物资储备，我们所有的医学装备的这个防疫物资都放在三楼，所有的病区的护士到三楼去取这个防疫物资，减少了我们楼外这个人给每一层推送防疫物资的感染风险，这是一个。再一个我们的信息保障组，就是要保障我们所有的信息畅通，我们在这个配送物资的时候呢，我们开始由我们总务科自己采购，后来我们就在我们医院综合收费平台上，在第三方超市进行那个协议供货，当第三方超市的这个协议供货的它的价格、品格和规格所有的生活物资，都跟我们医院的综合收费平台进行个嫁接，费用能够嫁接，我们代收代购，一分钱不加给他转出去，患者的话扫描付费，用最快的最便捷的这种购货方式，满足他们对生活物品的需求。这里物品特别多，这些患者基本上不做医疗治疗，主要是生活，从指甲剪到所有的这个生活的剃须刀等等的生活物品，非常的多。也就是说，我的体会是在疫情之下的综合物资保障必须建立一个快速反映的团队，这个团队的协作竞争非常重要，我们这六个部门的，我们建立了个疫情防控的物资群，大家在群里面就是呼之即响应，响应必能战，战能必胜。我觉得在这次的工作当中团结协作的精神是非常重要的，而且大家呢，那我在这个物资群里面，虽然是组长，我觉得就跟那个战斗一样，承担指挥员、司令员集于一身的作用，因为作为一个党委委员，我也经常在我们的群里面不断的给大家鼓舞士气，包括上级的精神的传达给大家，点赞，帮助大家共度难关吧，共同想办法，投身到一线，跟大家一起战斗，这是我的最深的体会。胡教授，我就说这么多。

J：胡教授，我补充两点啊，刚才夏总讲我们现在还有七八千万的家底。其实这个家底我再说出来呢，可能胡教授不敢相信，我们家底几乎是零。

H：我估计你没有家底，你欠一下药商的钱。这里先不说，等一会儿我再说一下我的想法。其实我晚上给他们开个会，其实还有一个想法，等一会我统一说一下，我现在提前说一下，包括张助理和夏总提供这句话，正是我们要做一事情，就把你们这些数据信息采集来，我们写一个政府建言，现在政府需要我们给省政府提供一些政策性的建议，那对建议的数据最好来自你们。我是第三方的说你们，你们自己说就是（不太好），我说就是（比较）好。这个一会再说。
