# Supplementary material for: Experience of rehabilitation management in public hospital after it was identified as designated rehabilitation hospital for COVID-19 patients: A qualitative study
Source: Front Public Health. 2022 Jul 26;10:919730. doi: 10.3389/fpubh.2022.919730 (PMC9362772; doi:10.3389/fpubh.2022.919730)
Supplement: Supplementary file 1 [file Data_Sheet_1.ZIP › Interview data/消化内科主任.docx]

W（武主任）：我就说一点点感受，就是因为可能我毕竟没进病区。嗯，教授好，是这样子，现在虽然没有进入病区，科里这些人员可能习惯了先给主任说有些事情看看能不能解决，能解决了这个事情就过去了。更多的反应就是说，进到病区里的医护人员，我觉得他们的心理压力比工作压力大。他们面对病人的时候，他第一个是要转换他的这个工作模式，以前是给病人看病，现在面临的病人是康复的病人，不是一个现在需要治疗某个脏器的问题，（病人）有这个身体的疾病，我去治疗疾病。现在针对的绝大部分的病人都是康复的病人，是已经治愈的病人，即便他有后遗的一些症状，我觉得这些症状可能一个是本身疾病有一些没有完全彻底恢复的一些症状，第二个可能是一些药物的后续反应，再一个可能我觉得很大一部分还是跟心理作用有关系。所以这个导致我们医护人员跟病人接触的时候，我们更多面临的是这个沟通的压力、交流的压力，而不是我们治疗的压力。还有一个就是现在的病人人人都拿手机，小孩都拿。其实我们在正常情况下，正常开展的时候，我们会多少限制一些住院病人使用手机，尤其在治疗期间。你身体有疾病的时候，我们到晚上要查房或者干啥都提醒一下，但是现在我就跟我们的医生和护士说，他想玩，就让他多玩一会儿，尽量不要限制他们，一个是让他有一个渠道释放他的这个情绪，转移他的注意力，如果再不让他有这么一个情绪的出口，可能我们医护人员面临的医患关系就会更紧张。因为毕竟他们从发病、隔离、治疗，可能都不止半个月的时间了啊，从前面被隔离或者管控或者密接或者什么，一直到核酸查出来到治疗，一直到出来到我们医院这里康复病区，可能都不止半个月的时间。这么长的时间，他们本身从开始得病，他们也面临社会上的压力，面临周围人的（压力）、信息被暴露的压力、心理的压力、疾病的压力，他们压力也很大，那么他们现在到康复病区了以后，我觉得他们可能确实有一些挑剔的心理，我觉得他们挑剔的心理可能不是针对这个疾病的，他们挑剔的心理可能针对后期我们给他服务，他觉得会有哪个地方做的不到位就很容易经成为一个情绪的激烈的交接点。那么我们现在就是尽量让我们的大夫更加小心翼翼一些，比平时给病人看病更加小心翼翼一些，说的话更软一点，态度更好一点，更主动一点，然后把这样的情绪安慰好，其实我觉得可能是更主要的。其他的就是在治疗方面，我觉得没有过多的压力，主要就是一个心理疏导，按部就班的去查房、舒缓压力，解决他出现那些问题，然后我觉得及时沟通、保障这方面的压力更大。再一个主要还是心理压力，我觉得医护人员的心理压力会比工作的压力更大，这个病人抖音、快手、头条，随时都可以用一个视频就发泄，稍微有一点点不满，我也不跟你交流，直接微博就发出来了，所以这个我们可能面临的很大一部分压力在这方面。这就是我的一点感受哈，其他没有太多。

J：已经做好了进入病区的准备了。胡教授，我们的同志基本把自己的一些工作和自己的体会都基本讲完了。

H：每个人说得很具体，也很详细，我觉得都比较实实在在。其实这样的会议我觉得还是应该给xx（人名，没听清）听一下，今天这个有录音了，我们的学生把它录下来。需要的话，我们可以把这个结果给他们有些人用什么方式来发给他们我们的一些情况，所以咱们再说吧。接下来这样吧，你们的情况我们清楚了，真的是大家都很辛苦，你们有些人的辛苦，我们是想象不来的，包括一个简单的核酸检测。
