# Supplementary material for: Experience of rehabilitation management in public hospital after it was identified as designated rehabilitation hospital for COVID-19 patients: A qualitative study
Source: Front Public Health. 2022 Jul 26;10:919730. doi: 10.3389/fpubh.2022.919730 (PMC9362772; doi:10.3389/fpubh.2022.919730)
Supplement: Supplementary file 1 [file Data_Sheet_1.ZIP › Interview data/纪委-负责医院人员管理.docx]

W（王书记）：胡教授，那来给你们这汇报一下我们人员管理的大概情况啊，因为这个人员管理，作为康复医院来说可能还没有具体的一个实施办法，我们从接受这个任务之后，一直也是按疫情防控办下达了一个指导意见来实施。总体我们医院具体做的工作，大概有三个大的部分。一个就是从医院层面，医院层面做的一个总目标就是零感染，所以说我们主要是以预防为主，做好人员监测，人员的健康监测。一个是从思想上，大家一定要有预防意识，这个思想意识要到位，我们医院及时开会，成立专班这些。第二个是从制度上，包括人员管理、闭环管理的这些制度上建立在从制度上预防，从召开视频会，包括还有这个感控的培训、宣讲，从制度上给大家预防，具体到我们从专班，从工作人员管理这个专班来预防，我们现在主要实施的有几个环节。第一个就是从对象上，昨天开会就是调整（对象），之前我们总认为是进了病区这个严格闭环管理的是主要的对象，昨天开会说是，前天晚上市上要求我们这些医院全部（工作人员）视为重点的观测监测对象。所以说我们现在将对象调整为全部医院的全部在岗人员，这个对象是明确的。其实我们前期对象明确之后，就是健康筛查，所有上岗人员必须做到健康筛查，这个省上是有明确的一个指导意见。首先就是流行病学的一个调查，新冠疫苗有没有全程接种，这个是最基础的。这些基本条件达到之后，做健康的复查，复查的内容就是现在所提的“1+3”——核酸检测、血常规、抗体检测以及CT检查，正常之后才允许上岗。如果是异常的话及时和医疗这口对接，及时调整岗位。正常之后进入第三个环节，就是日常的监测，主要是从重点人员的这些闭环的管理，提供食宿，酒店有专门的人员，或者管理整个酒店的闭环管理的人员，（我们）还专门制定了医院院感部门制定的闭环管理规定。实施中是各科室，从科室和个人日常上报之外，作为监测的酒店，这个具体酒店的返点人员还要同时在上报，属于是双管理，把这个健康监测一定要做到位。现在是根据人员专班，我们日常每一天都要统计，把异常人员再确认，是不是填错的，当然也有可能是有一些不填的。只要发现异常，所有人员是确认到人，及时处理，这是第三个环节。第四个就是我们在具体操作中的跟其他部门协作的这个区域分类管理。比如说，康复病区和核酸采集，还有一些酒店的保障这些都是按分工不同，把住宿的酒店都是严格分开的，不交叉，尽量做到工作的时候不串岗，轮班的时候，酒店封闭的时候，食宿安排，这些都是不交叉的，包括车辆也是不能交叉。第五个环节就是下一步的离岗管理，现在我们第一批人员，马上也就14天快到了。现在的指导意见来说的话是要在酒店隔离14天再等待下一步的安排。我们现在是计划按上级的部门的指导意见来随时调整。隔离期间还是要正常及时监测大家的健康情况，具体就是三个方面的预防。胡教授，您看我们这就是具体的从操作方面，可能手上只是指导意见，所以说没有一个具体的规范，我们也是从人员安排上是逐步调整，具体的实施过程中也是一步一步在摸索，如果说现在大家把这个经验一点一点积累一起来之后，各个康复医院可以互相有无，把一些经验互相总结给大家，也下发一些我们具体做法的一些要求或者规范，其他就没有什么。
