# Supplementary material for: Experience of rehabilitation management in public hospital after it was identified as designated rehabilitation hospital for COVID-19 patients: A qualitative study
Source: Front Public Health. 2022 Jul 26;10:919730. doi: 10.3389/fpubh.2022.919730 (PMC9362772; doi:10.3389/fpubh.2022.919730)
Supplement: Supplementary file 1 [file Data_Sheet_1.ZIP › Interview data/院长专访.docx]

**关于陕西省第四人民****医院被确定为新冠肺炎出院患者定点康复医院——对贾延院长的专访**

1. Q:将陕西省第四人民医院确定为新冠病人康复医院，医院都为此做了哪些准备？包括病区、人员、设施设备等；

A:我们是元月六号晚上接到的通知，要把我们医院定为新冠康复医院。当天我们就对这个决策做了一些简单的思考，思考到底应该怎么去做这个事情，怎么样才能做好这个事情。然后七号上午我们就召开了一个院党委扩大会啊，因为通过党组织来把这件事情通向我们的党委委员，向我们相关职能部门进行了通报，然后研究确定把我们医院作为新冠康复医院以后我们的准备工作该怎么做，这是七号上午的工作。除此之外，七号还有一个重要的任务，就是我们要把本院全部在院病人全部转出，七号时我们在院的病人还有二百二十五名，上午十点钟开完会以后，我们就开始动员每个科室，动员所有的患者，能够出院的就出院，能够转院的我们协助患者进行转院，截止到晚上十点钟，我们已经把所有二百二十五个患者全部转出成功，这其中有近八十名危重症患者，通过我们和其他兄弟医院对接，我们主动联系把这些危重患者通过120急救车和我们院的救护车安全转运到了能够接收他们的医院，顺利地完成了这次病房的统控工作。

晚上，我们连夜对病房进行了整理和消杀，这是对病房做的最基本的准备。然后七号晚上我们对我们的基本的流程——就是院感要求的基本的三区、两通道的流程，做了一个初步的确定。明确了我们的清洁区、半污染区和污染区的划分，这在院感上是非常关键的一个环节。在这块呢我们也做了充分的准备。八号一天工作的工作，我们继续对整个院区的工作环境进行了一个充分的准备。在整个院区，我们对接了运送患者的120的通道，对此做了一个相对的固定程序。然后按照传染病医院的三区、两通道的一个区别，把我们的患者的通道和我们内部职工的工作通道进行了相对的硬隔离，保证了除了我们在康复病区工作的医护人员以外，其他的工作人员不接触患者，这能够有效防止疾病的传染。

**以上是我们在硬件设施方面所做的一些准备工作。**

其次，在组织架构上，我们划分和安排了六个工作小组来分工完成本次任务。第一个是我们的综合协调组，综合协调组负责医院内部的对接以及和上级政府机构的对接工作。就比如说和指导我们工作的卫健委、防疫指挥部进行对接，把他们给的各项指导意见和措施落实到我们这个定点医院。在医院的对接就是对接病人如何转运的问题，除了各项对接工作之外，综合协调组，还要协调我们全院的医护人员吃、住、行的问题，因为按照要求所有进入康复病区的人员全部要闭环管理，所以这一点对我们来讲压力也是比较大的。然后综合协调组还要协调整个院区环境的管理。

第二个是医疗保障组，医疗保障组主要是负责对新冠康复患者的医疗救治、转运和康复治疗这方面的工作。除了这项工作外，它还承担着对危重症患者的急救工作。

第三是院感组。院感组在我们本次作为新冠定点康复医院中发挥很大的作用，因为新冠是个传染病，要防止传染病再感染，院感组的工作是非常重要的，而且国家给我们的任务和要求是我们院内感染要是零且必须保持在零，确保零感染这个任务也是非常重的。我们院感组也非常辛苦，对我们医院的大环境、工作流程和隔离病区的路线进行了严格的划分，也做了一些标识，再还有就是对我们的防护级别也给了非常重要的指导和建议。我们现在院内的个人防护和环境消杀工作都是做的非常好的，严格按照了应对新冠的各项要求和标准。

第四是物资保障组。一个是我们经费的保障。因为这次的新冠疫情对我们来讲，我们没有拿到专项经费，自己也没有去申领，全部都是由医院自己先行支付的，可以说是“受命于危难之时”，无条件地接下了这个任务。然后经费的保障由总会统筹安排，包括医院的资金运行、还有医疗设备和防护设备、防护设施的保障，当时只留给我们八号一天的准备时间，还是非常紧张的。除此之外，患者必备的生活用品，比如说水壶、洗脸盆啊、垃圾桶这些必备的物资，还有每人每天要配发的口罩，都由我们物资保障组来完成。除了物资以外，还得保证我们给患者用的药物。因为新冠患者对我们综合医院来讲，我们也是第一次接触这类患者的治疗。所以在药物上我们还要想办法要保证常规用药和新冠的特殊用药。再还有就是保证患者的饮食问题，我们合理安排好了患者的一日三餐，明确了送餐和患者就餐的时间、送餐的路径和送餐的方法，保证零接触的情况下尽量满足患者要求。对于饭菜的质量标准，我们也保证患者在这里一日三餐能够有一定的营养，比如每天有一袋牛奶、每天要有一个水果，这些东西我们都要对患者保障。

第五是人员管理组。人员管理组，这个“管理”主要是说管理我们医务人员，我们医务人员现在分类和过去我们常规的一些医院的分类不太一样，我们分为几大类，第一大类是进入新冠病区的，这叫直接接触患者的这一类人群，这个人群我们现在是准备了近三百名人员分两批进入，近四百名医护人员分两批去我们的隔离病区、康复病区，每一批就将近是两百人左右，所以人员的安排也是非常关键。再一个，我们这个人员进驻以前，按照国家卫健委和防控指挥部的要求，要对所有的人员进行健康体检、身体检查合格以后才能进入，如果检查有问题，我们还进行调整。所以在这块工作量也是非常大的。这是需要管理的一类人，还有一类人就是保障服务人员，就是不直接接触患者，但是也有可能进入到这个康复病区周边的工作人员，这些人员的管理也是非常重要的。再有一个就是这次有些身体不太适合在医院工作的，比如说有些怀孕的、有些在哺乳期的这些同志，还有些年龄大的同志，不太适合继续在医院进行闭环管理的人员，我们也得要有一个合理的安排，让大家能把手头的各项工作做好。虽然我们医院的主体业务工作短时间变了，但是开通的互联网医院这项工作，或者是给隔离酒店做服务工作，这些工作还是需要人的，所以我们要进行一个工作的分流啊，让大家都能有事干。还有一组人就是我们驻扎在隔离酒店需要管理的工作人员，我们现在有四家酒店来接收我们医护人员入住，因为我们是闭环管理嘛，这个要求是我们自己管好，安排了十多个人对四个酒店进行管理。

最后一个就是宣传组。宣传组主要负责报道和宣传我们的工作。除此之外，宣传组还要做好舆情控制的工作。

以上是我们六大工作小组的主要的任务。

除了六个组的任务之外，我们也还有两项工作在同步的进行。第一个是承担社会面的核酸检测任务，我们承担新城区，目前只剩下新城区的核酸检测，我们前段时间承担了雁塔区、未央区、灞桥区、新城区四个区域的核酸检测。在新城区和灞桥区我们是常驻，有两百六十多名同志支援这两个区的核酸检测，在雁塔区和未央区，我们先是在统一指挥下临时支援，每次也将近两百人，目前这个核酸检测队伍我们还没有完全撤回来，还有八十个人在外边来完成社会面的很多检测任务。

还有就是我们这次转型为康复医院，我们所有过去综合医院的业务全部暂停。暂停以后对我们院的影响是非常大的，因为我们的专业比较全，这些专业现在都停止了。因为在我们所在的区域内，我们是唯一一家三级医院，每年为周边群众提供医疗服务，门诊量达五十多万啊，我们这一停，对这一片区群众的就医问题影响还是挺大的。如果解封以后有大量的就诊需求，就过去的承接能力还可以应对，现在全部暂停以后，这些患者可能要到其他地方去，还有一些慢性病患者取药的问题和长期用药的问题都是需要解决的。所以我们最近正在加紧开通互联网医院——互联网诊疗这项业务。这一次希望能通过这次机会，把互联网医院的建设抓紧完善，很快也会线上开通网上咨询业务，现在每天都有近千人的咨询量，说明群众的需求还是挺旺盛的，所以这项工作要马上继续做好。这个互联网医院，可以说是我们医院有自主知识产权的。

现在回到我们新冠康复医院，医院总共编制床位是七百七十张，开放也是七百七十张，我们这次腾出的住院楼有十二层，能够开设近六百张床作为康复病房，实际开放可能有五百九十一张，能接受五百九十一个病人，十二个病区，这个都是标准建制的病床，设备比较齐全，基本的救治工作都可以完成。每一层楼可以进入病区的人员基本上是六名医生和十四名护士，就二十个人，分四班倒，每天四班。一共十二层，就是两百四十人，还有几十个保障人员。算上外围的工作人员，可能每天进入这个病区里边的人将近三百人左右。我们医院的医疗设备、设施这方面都是基本的设施，没有什么太大的增加，因为是康复病人，从诊疗规范上来讲，不需要有太多的治疗方案和手段，但是我们也做好了有小孩，特别是婴幼儿的一些预案。在十二个病区里边，有两个病区是特殊病区，一个病区是儿童病区，有五十张床，还有一个病区是危重症病区，也有五十张床。这个危重症病区有七张监护病房，可以去接受危重症患者，我们的ICU还有十五张床可以接受危重症患者，所以在这一块的救治能力呢我们是有保证的。而且对常见的急、危重症的救治是我们医院的一个特长，所以在这方面我们可以通过这些有效的工作让患者在新冠康复期能够对疾病、或者其他的基础性疾病有一个很好的救治，保证他们安安全全地来、安安全全地出，这是我们最基本的医疗服务。

2）医院对新冠康复人员将采取哪些康复方法？

A:康复病人现在基本的康复治疗是基于它是一个呼吸系统的疾病，是在这个基础上展开的。通过国家相关领域的专家给我们指导和建议，我们采取的治疗方案就是做呼吸功能的锻炼，就是每天要指导患者进行深吸气、深呼吸，还有俯卧位的呼吸，这种锻炼会加强他们肺功能的恢复。如果说有一些并发症或者有一些其他的疾病，我们可以对症治疗。

再还有就是康复期，有些患者如果有需求，我们可以根据患者的需求进行中医药的治疗。如果有一些患者心肺功能不太好，比如老年人他需要吸氧，我们也会进行一些氧气治疗，这是一个手段，主要还是针对呼吸系统疾病嘛。

而且目前我们接受的二十三名患者都是轻症，并没有重症，所以康复起来相对来说简单一些。

3）对新冠康复人员医院如何进行社会及心理方面辅导？

客观来讲，这方面其实我们并没有太多的经验。但是从我们医院来讲，我们会要求所有的医护人员给大家一些人文的关怀，因为毕竟是患病又在医院住院十多天，从封闭的区域又到一个封闭区域，患者在心理上可能会产生一些压力或者是其他负面的情绪。在这方面我们尽量。通过我们一些人文关怀来解决这些问题。

还有我们有心理科医生，我们拟通过互联网线上诊疗，对患者的心理问题进行一些沟通、交流、调解。心理科医生也是康复组的成员，会帮助给患者做一些疏导。

除了心理上的疏导外，我们也尽力在生活上给患者多一些关怀和照顾，比如说患者的生活方面，除了我们的医疗物资免费供应以外，还有一些生活物资供应怎么办？其实我们也在考虑，想通过我们和大型超市进行对接，然后直接转运给患者。第一、这可以保证我们生活物资的质量；第二、价格透明；第三、这样也减少了我们在外面采购的环节，患者满意度能高一些。还有就是我们医院所有的病区，过去开放过热水，不能洗澡，我们这次跟供热公司进行沟通，二十四小时供应热水，可以解决患者的清洁问题。所以在这些生活方面得到保证以后，我想患者在这里过得不至于说很压抑。

还有一方面，已经给我们护理部已经讲了，可以在患者中尝试着进行护进行多一点的沟通、交流，然后采取一些形式多样的一些活动，让大家能够投入参与到活动中，能够在这个十几天的治疗过程中都是比较愉快的吧。


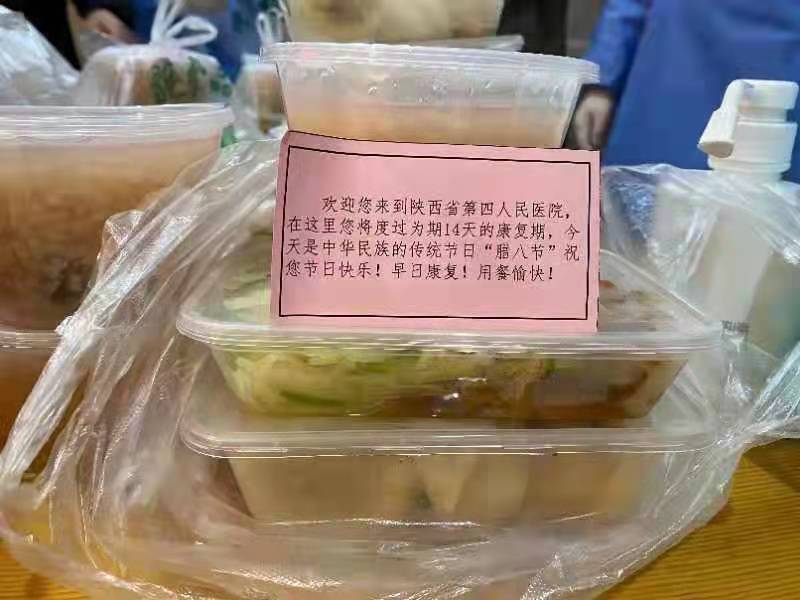


**（腊八节对患者的问候）**

4）对新冠康复人员的日常生活如何安排？

因为对新冠康复患者的要求是不出隔离病房，所以他们的生活基本上全天候都在病房里边。然后我们早中晚都会按时送餐，生活其他方面的需求主要就是物资采买和个人卫生清洁的问题，这些问题的话通过我们刚刚所讲的超市代购和热水供应可以解决。至于其他的生活需求和生活安排，这个要根据病人自己的情况进行自主安排，可能会在病房做一些康复训练或者是休息娱乐之类的，病人还是比较有自主性的。

5）确定康复出院的标准是什么？

这个问题其实国家是有明确标准的，就是需要在二十四小时间隔完成两次核酸检测，两次核酸检测均为阴性，然后进行双采（就是鼻咽试纸和口咽试纸都采），最后一次双采采完以后，均为阴性，且没有任何发烧发热等症状就可以出院，这是目前所采用的标准。

6）职工的态度的行动如何？

其实，我个人是对我们的全体职工道一句感谢的。我们医院的职工在接受这次任务的时候，表现得非常积极、对于我们的各项工作也非常支持。前期我们其实并没有做大量的动员，但是职工能够主动地参与到这项任务里面，其实让我非常感动。

因为我们接这个任务其实是属于“奉命于危难之间”，六号接受任务，九号就开始接受患者了，时间是非常紧急的，绝对不是单靠一个人或者一个小组的力量就可以做好全部的准备工作，是全体职工的积极支持和果断行动才推动了我们各项举措的快速高效开展。这也让我看到了在一些“特殊”时期，大家的心愿是相同的，心灵是相通的，整个医院的凝聚力是非常强的，这绝对离不开每一位职工的支持和付出，无论是在病区的医护人员，还是做后勤保障或者在其他岗位上继续完成任务的职工，都让人很感动，是每个人都承担好了自己的个人责任，才能加总在一起让我们医院承担得起这次作为新冠康复定点医院的社会责任。

其实对于本次的这项任务，在战略上，我们是既把它当作一项上级交付必须高质量完成的政治任务，又把它当作一项服务群众反馈奉献的社会任务，同时还把它当作了我们实现自我改革和转型的内部任务。所以说，这个任务对我们来讲是具有政治性、社会公益性和自我改革性这三重属性的，因此全院上下对此也非常重视。我们在承接这项任务做系列决策的同时，是对过去的管理进行了系统复盘、深刻反思和经验总结的，吸取了在过往医院管理上一些失败的经验教训，同时也积极向行业优秀的标杆单位进行了取经学习，恰逢也是新年之际，也希望能够借这次机会做好院内的整顿、整改和转型发展。我们也希望能够在高质量高效率地完成本次新冠病人康复救治任务之后，继续“趁热打铁”，凝聚起医院全体职工的“干劲”和“拼劲”，做好医院的后续转型和发展工作，凸显出我们的“公益性”特色，办好真正让人民满意、真正为人民服务的“一流好医院”！
