# Supplementary material for: Experience of rehabilitation management in public hospital after it was identified as designated rehabilitation hospital for COVID-19 patients: A qualitative study
Source: Front Public Health. 2022 Jul 26;10:919730. doi: 10.3389/fpubh.2022.919730 (PMC9362772; doi:10.3389/fpubh.2022.919730)
Supplement: Supplementary file 1 [file Data_Sheet_1.ZIP › Interview data/麻醉科主任-医疗专家组组长.docx]

Q（乔主任）：胡老师好！我简单说一下我们医疗救治组这次主要的（任务），主要是负责医院在院的584名康复患者疾病的一个治疗指导。第二个是做好我们病区和医疗管理部门之间的一个衔接工作。第三个就是如果病区的这584名患者出现紧急的意外情况，比如说他可能发生后心电骤停的，比如说还是其他什么意外情况，我们及时进入病区进行一个抢救。我们这个组是24小时在岗24小时待命，病区发生任何状况，他随时会打电话，先电话沟通，我们以远程会诊为主，那么在电话沟通的过程中我们给他一个治疗方案，然后他在执行的过程中，如果这个患者的症状不缓解，有任何问题，我们再随时进一步的沟通下一步的治疗方案。所以说我们其实在康复患者整个过程中，其实发现的问题是，这个病人本身的疾病并不是说非常让病人难以接受的，主要可能还是一个心理的作用。在这个疾病过程中，它主要表现就是一个干咳，也就是新冠肺炎后期肺上的问题，那么这个干咳症状其实一般不需要处理，但是这个病人在住院这一过程中，如果不处理的话，他确实会认为我们医护人员不尽职不尽责，所以说在这种情况我们多半跟他说要多喝水，然后必要时吸氧，但是患者还是要求吃药，所以我们必要时开一些甘草片、肺力咳之类的，然后其实他不吃药的情况，他症状也在逐渐缓解，但是我们必须要给他开药，否则他心里认为我们医护人员不重视，所以这种情况下，在吃药的过程中的症状也是逐渐缓解的。

第二个，其实这个病人的合并症是一个问题，就是第一个病症状态，整个病人基本上有时候体温在36.9度—37.3度之间，甚至是37.5度这样一个状态，那么有的患者可能是要求我们必须要做核酸检测，但是按照目前国家的要求来说，目前这一部分病人是不需要做核酸检测的，所以这部分我们的医务人员大量在做这个解释工作，我们也在不断的做解释工作。当然，如果说他的体温持续上升的话，我这一块核酸检测是应该立即进行的。所以说，其实在整个过程中病人心理方面存在的主要问题是，他本身对疾病认识不足，对我们的隔离政策的不理解，担心愈后从而产生的一个焦虑情绪。那么针对这类问题，其实我觉得，我们医务人员在跟病人沟通的过程中，吃药是一个方面，但最重要的是我们医护人员要加强对患者的政策解释和宣讲，以倾听和鼓励为主，对疾病产生的焦虑主要需要我们医务人员对疾病知识进行宣讲。所以说，我们医务人员对这一块的疾病认识和政策要充分的了解。还有一个就是因为病人不断地去换环境，第一次可能是在其他医院，那么第二次的14天又在这个医院，可能出去之后又到其他地方，所以说，他因为这个环境不适应而产生的失眠问题也是需要我们去不断处理的，那么，在这个过程中，我们医院也开展了一个线上的心理咨询，患者出现了一个任何心理的问题，我们线上的一个主任医师也是不断地在讲解。那么，我觉得这个后期存在的问题是，患者出院后可能会存在他的社会适应能力下降的问题。在我们这八天的时间里，跟病人沟通过程中，有的病人是产生了自卑的心情，他觉得如果出院之后，可能会远离他的生活圈子或者团队，所以说这个可能需要我们全社会积极帮助新冠肺炎患者重新树立生活和工作的热情，那么在这种情况我们也在想，通过电话、微信的方式增加沟通交流，让这部分患者充分认识到没有被社会抛弃。这就是我们医疗救治组在这个患者救治过程中简单会总的几点，胡教授。

H：好好，谢谢！辛苦了！
